# Supplementary material for: Epigenetic editing balances TCR suppression and persistence in CAR T cells
Source: Mol Ther Adv. 2026 Mar 6;34(2):201712. doi: 10.1016/j.omta.2026.201712 (PMC13148917; doi:10.1016/j.omta.2026.201712)
Supplement: Document S1. Figures S1–S3 and Tables S1–S4 [file mmc1.pdf]

OMTA, Volume *34*

Supplemental information

Epigenetic editing balances TCR suppression and persistence in CAR T cells

Pascal Y. Schönberg, Ángela Muñoz-Ovalle, Haidy A. Saleh, Eugenia Crespo, Robert Kuhnert, Susanne Michen, Liliana Loureiro, Achim Temme, Anja Feldmann, and Frank Buchholz

Supplemental Information

Table S1: sgRNA sequences

sgRNA name	Sequence (5' – 3')	Source
sgCD3ε-8	ACAGGAAGTAGAGAGGCCTC	this study
sgCD3ε-9	AGCTGTCCAGACCATGTAGT	this study
sgCD3ε-10	TCTGTGTGGTGGATCTGGCA	this study
sgCD3ε-11	GTGATAGTGTCAAAGTCTGC	this study
sgCD3ε-12	TACCTTAGAGGGTCGTTTTG	this study
sgCD3ε-13	ACTGTACTGTAGTTATATGC	this study

Table S2: Designed zinc finger proteins

Zinc finger name	Predicted DNA target Sequence (5' – 3')	Designed protein sequence	Source
ZnfCD3ε-1	TGTATTTTTCATGGC CCCTCC	FACDICGRKFAHKRSLLDHTRIHTGEKPFACDICGRKFADRS TLRGHTKIHTQRPQIPPKPFACDICGRKFARKDALLNHTRIH TGEKPFACDICGRKFATSSHLGHHTKIHTQRPQIPPKPFACDI CGRKFAWKHVLLNHTRIHTGEKPFACDICGRKFAWLSSLSA HTKIHLRGS	this study
ZnfCD3ε-3	TCAACCAGTCCAGG TGGAGGC	FACDICGRKFARKYHLSQHTRIHTGEKPFACDICGRKFALRW RLNYHTKIHTQRPQIPPKPFACDICGRKFARKWNLLTHTRIH TGEKPFACDICGRKFADPSSLRRHTKIHTQRPQIPPKPFACDI CGRKFADRSTLRNHTRIHTGEKPFACDICGRKFAQSSSLYKH TKIHLRGS	this study
ZnfCD3ε-5	CCAACCTACTACATG GTCTGG	FACDICGRKFARVDVLLDHTRIHTGEKPFACDICGRKFATSS HLSRHTKIHTQRPQIPPKPFACDICGRKFAQSGTLYNHTRIHT GEKPFACDICGRKFALKQTLQRHTKIHTQRPQIPPKPFACDIC GRKFADRSTLRNHTRIHTGEKPFACDICGRKFARRGDLNRH TKIHLRGS	this study
ZnfCD3ε-6	GGAAACGTTCAAGG CAGAGGC	FACDICGRKFARKYHLSQHTRIHTGEKPFACDICGRKFARK WNLRMHTKIHTQRPQIPPKPFACDICGRKFARKDHLKNHTR IHTGEKPFACDICGRKFATSSHLCHHTKIHTQRPQIPPKPFAC DICGRKFADPSNLNRHTRIHTGEKPFACDICGRKFAQRAHLT RHTKIHLRGS	this study
ZnfCD3ε-7	GGGGAGGAGGGTTT CTGAACC	FACDICGRKFADPSNLNRHTRIHTGEKPFACDICGRKFARRS TLRSHTKIHTQRPQIPPKPFACDICGRKFAYKHVLRNHTRIHT GEKPFACDICGRKFAQLYYLSRHTKIHTQRPQIPPKPFACDIC GRKFARKFNLLRHTRIHTGEKPFACDICGRKFARRYSLRCHT KIHLRGS	this study
ZnfCD3ε-10	CAGCCTTCCTAGAAG GCCAAA	FACDICGRKFAQKGNLNSHTRIHTGEKPFACDICGRKFAFRS KLRRHTKIHTQRPQIPPKPFACDICGRKFAQKVHLLNHTRIH TGEKPFACDICGRKFAWKIDLVRHTKIHTQRPQIPPKPFACDI CGRKFALKQTLQSHTRIHTGEKPFACDICGRKFARKWNLSM HTKIHLRGS	this study

Table S3: List of misregulated genes of CD3 epigenetic editing samples from RNAseq

Donor 1			Donor 2		
**Gene**	**log2FoldChange**	**p-value (adj.)**	**Gene**	**log2FoldChange**	**p-value (adj.)**
CD3E	-3,4948277	1,00E-116	CD3E	-2,7924095	0
EGR1	-2,0981087	8,20E-23	EGR1	-1,5674684	0,00141693
RUNDC3A-AS1	3,84431362	4,16E-20	RUNDC3A-AS1	2,40972077	3,06E-19
PSD	-1,8270684	0,04823351	IL3	-4,5201647	0,01561663
PDE4A	-1,7798847	2,60E-06			
EPN2	-1,9848694	0,01650199			
ATP12A	-1,7306593	2,91E-05			
PLXNA2	2,00384955	0,00052638			
MCAM	-1,6447798	0,00809591			

FGFR1	-2,0384521	5,57E-34			
COL5A3	-1,780379	0,00211456			
P2RX5	-1,6489737	1,56E-05			
PITPNM3	-1,9364153	0,0305436			
SORBS1	1,60122999	0,04028409			
MLC1	-1,9297779	1,27E-10			
LYL1	-2,3502757	0,00251292			
PLPPR2	-1,6743729	0,00729399			
ISYNA1	-2,2138477	2,41E-06			
HSPB1	-1,5740675	0,00631777			
AEBP1	-1,5615849	0,00020769			
NPDC1	-1,8593475	0,00013883			
CCL2	3,20270636	5,41E-05			
PCSK4	-1,9529394	0,02902458			
HSD11B1	1,5267058	0,03857889			
EGR1	-2,0981087	8,20E-23			
NUDT10	-2,0432582	0,00030984			
NRSN2	-1,670366	0,00631777			
GAD1	-1,5362809	0,00080276			
CABLES1	-1,7465919	0,00071047			
PCDH8	-1,8443302	5,06E-05			
SCN2A	2,5433992	4,09E-05			
CTSV	1,59538867	0,00065765			
FXYD2	1,61342572	0,00885732			
STON2	1,5531341	0,02466638			
RHOB	-1,8485461	0,03412551			
HES6	-1,5599005	0,00743889			
PLK2	2,0868706	1,01E-05			
DDX4	1,59599097	0,00296725			
CLGN	2,18332199	0,01013801			
SLC5A10	-1,8001748	0,00396074			
NRGN	-2,1098367	0,02195611			
DUSP2	-2,3916972	1,69E-10			
RHPN1	-1,6873771	0,04067628			
NBL1	-1,7292823	0,00151276			
TGFA	1,78938641	0,01855837			
NUAK2	-1,6612045	0,00654461			
COL1A2	1,55575261	0,03364124			
CORO6	-1,7536526	0,00325154			
TNXB	-1,6036959	0,04075605			
DSEL	1,52808341	8,56E-05			
ODAPH	1,78969388	0,00016548			
MARCKSL1	-1,8011079	5,57E-14			
TUBB6	-1,6583386	0,01013801			
SPHK1	-2,0955645	0,02630609			
METRNL	-2,0491371	0,01013801			
RNF212	1,72396032	0,0215462			
EGR3	-2,0514156	0,00049488			
LINC03040	-1,8327786	1,11E-05			
TNFSF15	2,60338448	1,09E-20			
RIMBP3C	-1,5458867	0,01690038			
PDE6G	-1,7573002	9,85E-05			
IFIT1	1,66261324	0,018333			
GAL3ST4	-1,6478281	5,33E-07			
KANK2	-2,1903606	0,00038556			
DLL1	-1,9269118	0,03039918			
MATK	-1,5604297	0,00966267			
IGHM	-1,7938412	0,00029578			
PLXNA4	1,93363359	2,02E-24			
MIR503HG	-1,6563988	0,04779445			
CYP2D8P	-1,9354755	0,04646615			
SGO1-AS1	-1,8027476	0,00342841			
FTH1P23	1,51909084	0,00944042			
SCARF2	-1,9057404	0,02091798			
CHD1-DT	1,62402299	0,01650199			
PURPL	2,5964203	1,08E-17			
LINC03066	-1,9665217	0,00043807			

CIRBP-AS1	-1,5186198	0,03840965			
TIMP2	-1,5603573	5,69E-05			
RIMBP3B	-1,659366	0,00737002			
RIMBP3	-1,8826161	0,0215462			
CCL3	-1,5604053	0,00030893			
GIPR	-1,6583424	3,03E-06			

Table S4: In silico predicted off-target binding sites of sgCD3ε-9

Chromosome	start	end	strand	MM	target_seq	position	gene
chr11	118304525	118304547	-	0	AGCTGTCCAGACCATGTAGT	Exonic	CD3E
chr8	33333326	33333348	+	4	AGGAGGTCAGACCATGTAGT	Intergenic	RNU6-528P
chr2	124917153	124917175	-	3	AGTTGGCCAGAGCATGTAGT	Intergenic	CNTP5
chr2	170863794	170863816	+	3	CACTGTCCAGACCTTGTAGT	Intergenic	GAD1
chr12	93676480	93676502	-	4	GGATGTCTAGAGCATGTAGT	Intergenic	CRADD
chr8	84720378	84720400	+	4	AAATGTCAAGAACATGTAGT	Intronic	RALYL
chr16	77962234	77962256	+	4	AGGGGTCCTGCCCATGTAGT	Intronic	VAT1L
chr3	37496754	37496776	-	4	AGCTGAAAATACCATGTAGT	Intronic	ITGA9
chrX	82111638	82111660	-	4	AGATGCCTAGACTATGTAGT	Intergenic	
chr5	138809578	138809600	+	4	GGCTGTACACATCATGTAGT	Intronic	CTN1
chr18	6836474	6836496	+	4	ATCTGTGCAGTTCATGTAGT	Intronic	ARHGAP28
chr2	12179051	12179073	+	4	AACTGTTCAGGACATGTAGT	Intronic	MIR3681HG
chr6	37882708	37882730	-	4	TGCAGCCCAGACCAGGTAGT	Intronic	RNU1-87P
chr14	103883469	103883491	-	4	GGCTCTCCAGGCCCTGTAGT	Intergenic	CTD-2134A5.4
chr8	39502547	39502569	-	4	AGCTTTTCATACCTTGTAGT	Intronic	ADAM3A
chr16	47777142	47777164	+	4	AACTGTGCAGAGCCTGTAGT	Intronic	CTB-55P19.1
chr4	137092461	137092483	-	4	CTCTCTCCAGACCATGCAGT	Intronic	RP11-138I17.1
chr1	245360680	245360702	+	4	CACTGTCCATACCATCTAGT	Intronic	KIF26B
chr6	63943963	63943985	+	4	TGCTGTGAAGACCATCTAGT	Intronic	EYS
chr10	75138183	75138205	+	4	AGCTTTTCAGACTGTGTAGT	Intronic	SAMD8
chrX	40178387	40178409	+	4	AGATGTTCATACCATATAGT	Intergenic	BCOR
chr10	112686703	112686725	-	4	TTCTGTCCAGAGCATATAGT	Intronic	RP11-25C19.3
chr10	117465139	117465161	+	4	CTGTGTCCAGACCATGTGGT	Intronic	EMX2OS
chr10	85712241	85712263	-	4	ACCTGTACAGAGCATATAGT	Intronic	RP11-93H12.3
chr2	11935562	11935584	+	4	TGCAGTCCAGTCCATGAAGT	Intronic	MIR3681HG
chr7	55736766	55736788	+	4	CCCTGCCCAGACCATGTACT	Exonic	CICP11
chr7	51382271	51382293	+	4	CCCTGCCCAGACCATGTACT	Exonic	CICP17
chr7	55798021	55798043	+	4	CCCTGCCCAGACCATGTACT	Exonic	RP11-419M24.2
chr7	128658782	128658804	-	4	CCCTGCCCAGACCATGTACT	Exonic	CICP14
chr7	39798389	39798411	-	4	CCCTGCCCAGACCATGTACT	Intergenic	CICP22
chrX	30698096	30698118	+	4	ACCTTTCCAGACCAGTTAGT	Intronic	RP11-242C19.2
chr1	53091887	53091909	-	4	AGTTGTGCAAACCATGTCGT	Intronic	SLC1A7
chr5	168975261	168975283	+	4	GGCTCTCCAGAGCATGTGGT	Intronic	CTB-174D11.2
chrX	55163432	55163454	+	4	AGCAATGCAGACCATGTATT	Intergenic	FAM104B
chrX	19325186	19325208	-	4	TGCTGTACAGACCCTGTTGT	Intergenic	PDHA1
chr3	112202780	112202802	-	4	ACCTGACCAGACCATCAAGT	Intronic	SLC9C1
chr2	219413294	219413316	+	4	AGGTGCCCAGACCATTCAGT	Intergenic	DES
chr13	56745239	56745261	-	4	AGTTGCCCAGAACATGTATT	Intergenic	
chr3	149323421	149323443	-	4	ATCTGTGCAGAGCATGTACT	Intronic	TM4SF18
chr10	37967726	37967748	+	4	GGCTGTCAAGACCATTCAGT	Intronic	ZNF25
chr3	142321429	142321451	-	4	AGTTGTCAAGACCATTCAGT	Intronic	XRN1
chr2	169085101	169085123	-	4	AGGTGTCAAGAACATGTATT	Intronic	DHRS9
chr3	84639100	84639122	-	4	GGCTGTAGAGACCATGTAGC	Exonic	LINC00971
chr13	34255433	34255455	+	4	ACCTGTGCAGACAATGTAAT	Intergenic	RP11-282M24.1
chr5	75371301	75371323	-	4	AGTTGTACAGACGATGTATT	Exonic	COL4A3BP
chr22	26805247	26805269	+	4	AGATCTCCAGACCATGGACT	Intergenic	MIATNB
chr3	116335988	116336010	+	4	GGCTGTCAAGACCATGCAAT	Intronic	LSAMP-AS1
chrX	107272279	107272301	+	4	AGATGTCAAGACCATGCAAT	Intergenic	MYCLP1
chr1	234647197	234647219	-	4	AGCTTTCGAGACCATGAATT	Exonic	RP4-781K5.9
chr16	73132574	73132596	-	4	AGCAGACCAGACCATGAAGC	Intronic	C16orf47

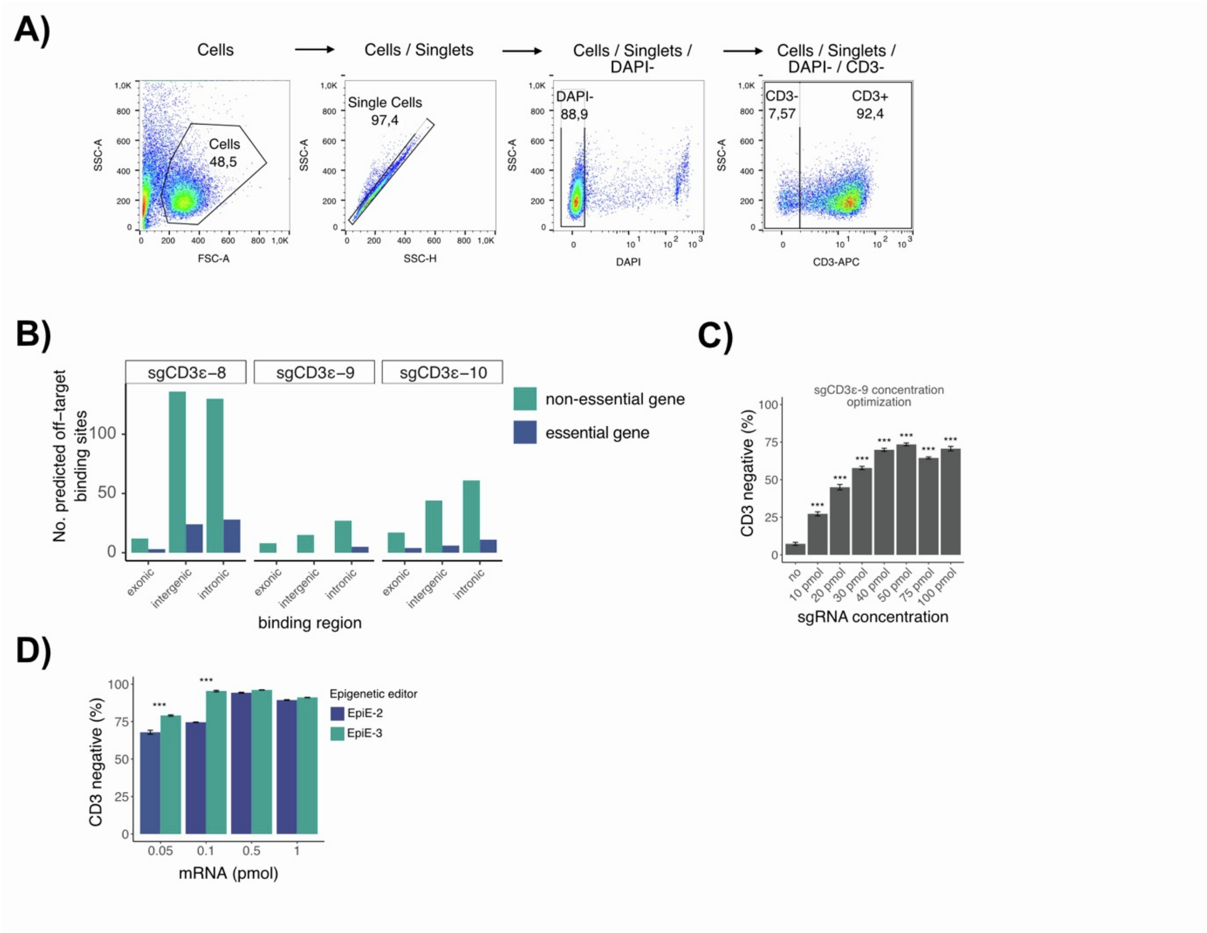

Figure S1: Gating strategy, sgRNA and epigenetic editor optimization

(A) Gating strategy of flow cytometry data to calculate CD3 negative percentage. (B) The number of predicted off-target binding sites in the human genome for the three functional CD3E-targeting sgRNAs is illustrated. Binding sites are specified by the binding region respective to the most proximate gene (exonic, intergenic, intronic) and genes are specified in different colors for their essentiality in human cells according to the Database of Essential Genes (DEG). (C) Optimization of the sgCD3ε-9 concentration for CD3ε silencing with EpiE-3 in Jurkat cells 4 days post transfection (p-values compared to “no” control sample: ***< 0.001). (D) Comparison of the epigenetic editors EpiE-2 and EpiE-3 at low mRNA concentrations in Jurkat cells 4 days post transfection (p-values compared to “EpiE-2” sample at the respective mRNA concentration: ***< 0.001).

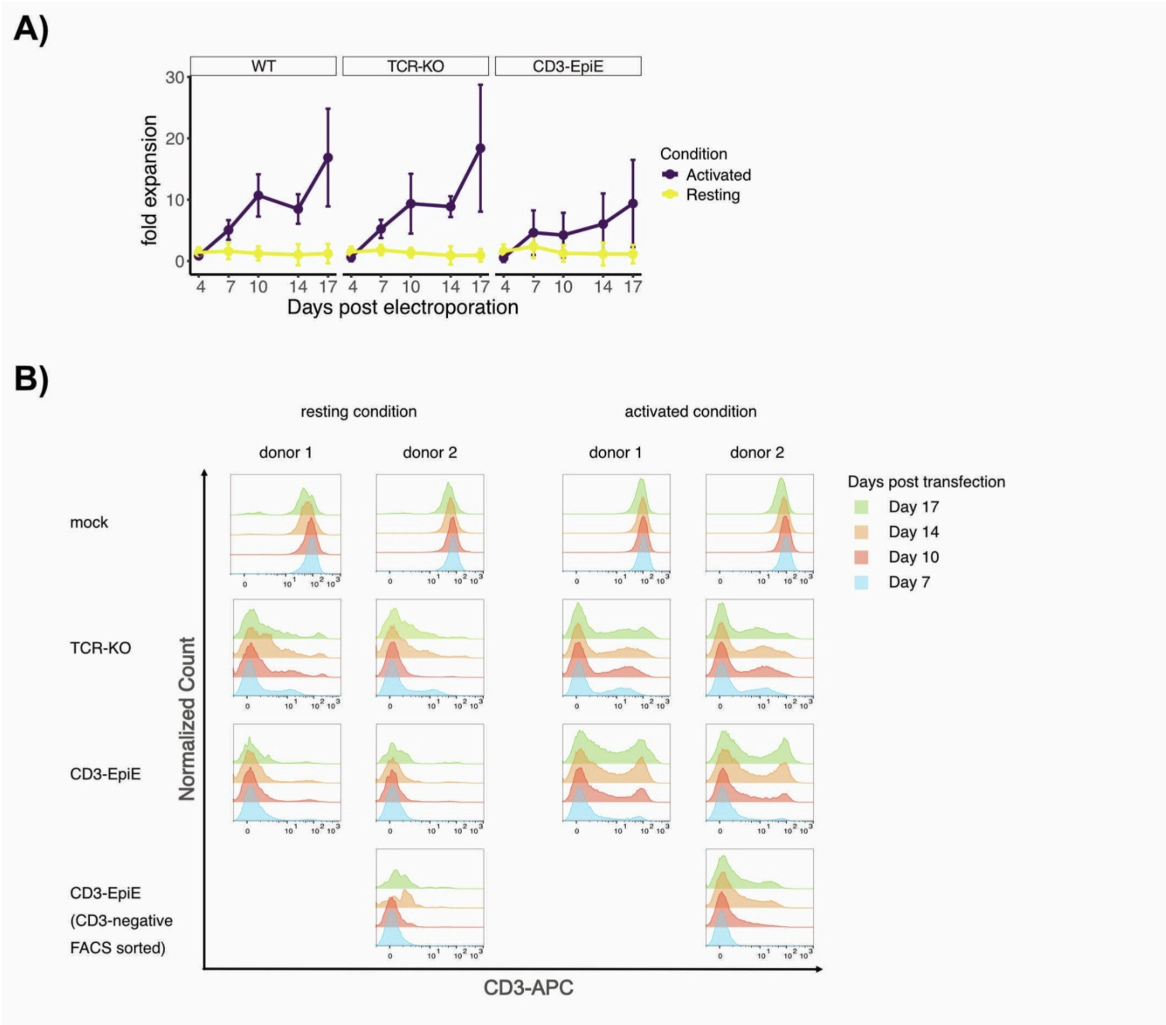

Figure S2: Expansion of primary T cells in resting and activated condition.
(A) Lineplot represents the fold expansion from the starting number of 1 mio. T cells over time in the resting (yellow) and activated (purple) condition over time for all three samples. The experiments was conducted on T cells from two individual donors (n=2). Dots and connecting lines represent the average fold expansion of all three donors and errorbars represent their standard deviation. (B) Flow cytometry histograms for CD3 expression under resting and activated condition for primary T cells from two independent donors. The editing conditions are depicted on the left, activation conditions on top, and timepoints for each sample indicated by different colored histograms in the same plot.

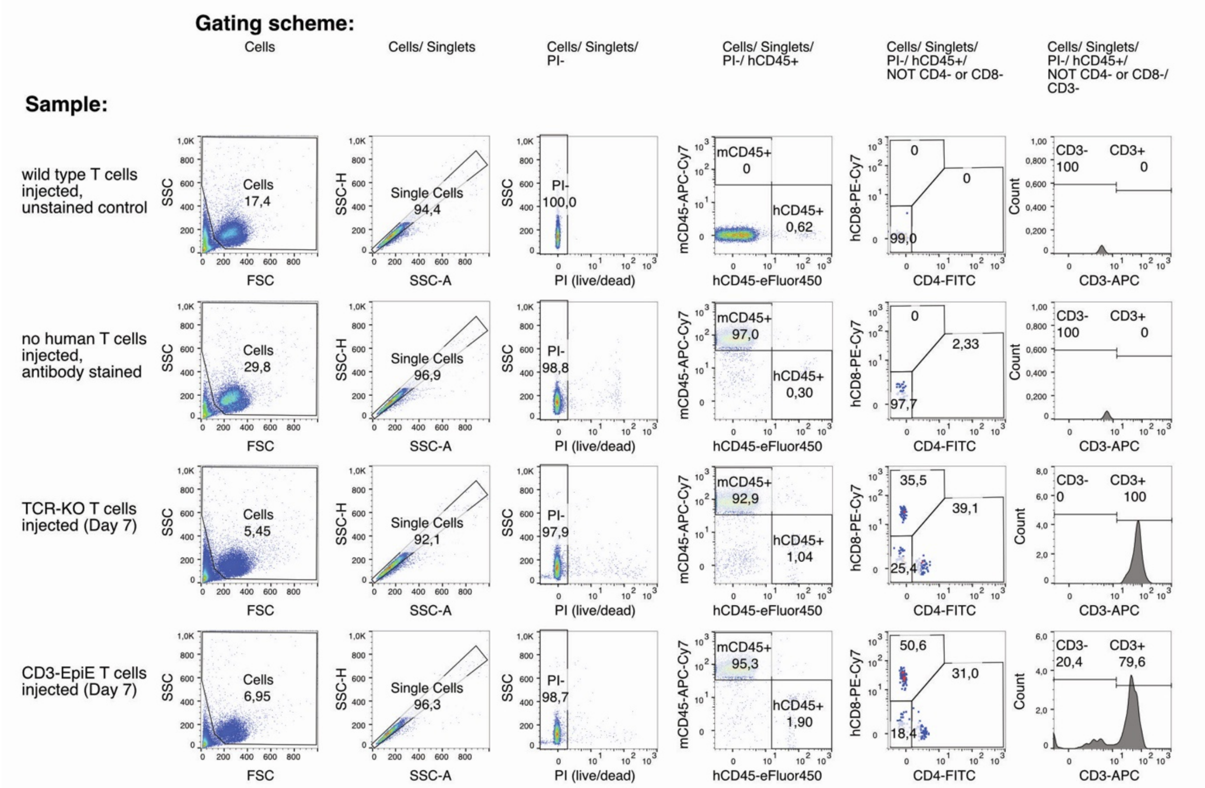

Figure S3: Gating strategy of mouse peripheral blood samples

Gating scheme for the calculation of chimerism ratio and CD3 negative T cells is shown for a unstained control sample, a control sample of peripheral blood without injected human T cells, a sample of injected TCR-KO T cells and injected CD3-EpiE T cells. Chimerism ratio was calculated as the ratio of viable mCD45^{+} to (hCD45^{+}/ CD4^{+} and CD8^{+}) cells. The human T cell CD3 negative percentage was estimated on gated Cells/Singlets/PI-/hCD45^{+}/CD4^{+} and CD8^{+} cells.
