# Supplementary material for: Epigenetic editing balances TCR suppression and persistence in CAR T cells
Source: Mol Ther Adv. 2026 Mar 6;34(2):201712. doi: 10.1016/j.omta.2026.201712 (PMC13148917; doi:10.1016/j.omta.2026.201712)
Supplement: Document S2. Article plus supplemental information [file mmc2.pdf]

Molecular Therapy
Advances
Brief report

Epigenetic editing balances TCR suppression and persistence in CAR T cells

Pascal Y. Schönberg,[1,7] Ángela Muñoz-Ovalle,[1,8] Haidy A. Saleh,[1] Eugenia Crespo,[2] Robert Kuhnert,[6] Susanne Michen,[6] Liliana Loureiro,[2] Achim Temme,[3,4,5,6] Anja Feldmann,[2,3,4,5] and Frank Buchholz[1,3,4,5]

[1]Medical Systems Biology, Faculty of Medicine Carl Gustav Carus, TU Dresden, Dresden, Germany; [2]Institute of Radiopharmaceutical Cancer Research, Helmholtz-Zentrum Dresden Rossendorf (HZDR), Dresden, Germany; [3]National Center for Tumor Diseases (NCT/UCC), Dresden, Germany; [4]German Cancer Research Center (DKFZ), Heidelberg, Germany; [5]German Cancer Consortium (DKTK), Dresden, Germany; [6]Department of Neurosurgery, Section Experimental Neurosurgery/Tumor Immunology, University Hospital Carl Gustav Carus, Technical University Dresden, Dresden, Germany

Allogeneic chimeric antigen receptor (CAR) T cell therapies offer a scalable, off-the-shelf option for cancer treatment, but their clinical use is limited by the risk of graft-versus-host disease (GvHD), mediated by the endogenous T cell receptor (TCR). Conventional strategies to eliminate TCR expression rely on genome editing tools such as CRISPR-Cas9 or base editing, which introduce permanent DNA changes and pose safety concerns. Here, we present an epigenetic editing approach that enables efficient, specific, and reversible silencing of the CD3ε gene, a critical component of the TCR complex, without altering the genome. We systematically optimized the epigenetic editor and guide RNA in a cell line and achieved robust TCR silencing in primary T and CAR T cells while preserving CAR expression, activation, and effector function. Transcriptome analysis confirmed minimal off-target effects. *In vivo* observations suggest the epigenetically silenced T cells to prevent GvHD while persisting longer than TCR-knockout cells, supporting the notion that transient TCR suppression may help balance safety and long-term efficacy. Our findings establish epigenetic editing as a non-genotoxic alternative to genome editing, offering a flexible and safer route to generate next-generation allogeneic CAR T cells.

INTRODUCTION

Chimeric antigen receptor (CAR) T cell therapies have revolutionized cancer treatment, but current autologous approaches require time- and cost-intensive manufacturing processes. Allogeneic CAR T cells derived from healthy donors offer scalability and rapid availability but are limited by the risk of graft-versus-host disease (GvHD), primarily mediated by the endogenous T cell receptor (TCR). Gene-editing strategies that knock out TCR genes using CRISPR-Cas9, base, or prime editors reduce GvHD risk but introduce concerns around genomic instability, off-target effects, and chromosomal rearrangements.[1–3] Moreover, recent studies have reported reduced *in vivo* persistence of TCR knockout (KO) CAR T cells, likely due to loss of TCR-mediated tonic signaling critical for long-term survival.[4]

Epigenetic editing provides a programmable alternative to nuclease-based gene disruption by modulating chromatin states without introducing permanent genomic alterations. Prototypic platforms such as CRISPRoff employ a nuclease-dead Cas9 (dCas9) fused to repressive effector domains, including Kruppel associated box (KRAB) and DNA methyltransferases such as DNMT3A, to direct silencing machineries to defined genomic loci.[5] These editors establish repressive chromatin features, such as H3K9me3 deposition and promoter DNA methylation, resulting in robust transcriptional repression. While this approach is non-genotoxic in that it does not create DNA breaks, potential risks include off-target binding of the editor and unintended transcriptional changes.[6,7] A distinguishing feature of epigenetic editing is its reversibility: depending on the specific marks deposited and the underlying chromatin context, silencing can be long-lasting yet reversible through targeted activation, or it can gradually diminish through cell division as repressive marks are passively lost.[8,9] This dynamic behavior may be advantageous for certain allogeneic T cell therapy applications, where transient TCR suppression could mitigate early alloreactivity and reduce acute GvHD risk, while subsequent re-expression might support long-term persistence and function *in vivo*.

Here, we present an optimized epigenetic editing platform for efficient TCR complex silencing in primary CAR T cells. This strategy offers a nuclease-free alternative to gene KO and may simultaneously address limitations in persistence for allogeneic CAR T therapy.

RESULTS

To disrupt TCR expression effectively, we focused on silencing CD3ε, a critical component of the TCR complex whose loss prevents the entire receptor assembly.[10,11] This strategy circumvents

Received 3 September 2025; accepted 3 March 2026;
https://doi.org/10.1016/j.omta.2026.201712.

[7]Present address: Seamless Therapeutics GmbH, Dresden, Germany

[8]Present address: Institute of Pharmacology, Heidelberg University, Heidelberg, Germany

Correspondence: Medical Systems Biology, Faculty of Medicine Carl Gustav Carus, TU Dresden, Dresden, Germany.
E-mail: frank.buchholz@tu-dresden.de

© 2026 The Author(s). Published by Elsevier Inc. on behalf of The American Society of Gene and Cell Therapy. This is an open access article under the CC BY license (http://creativecommons.org/licenses/by/4.0/).

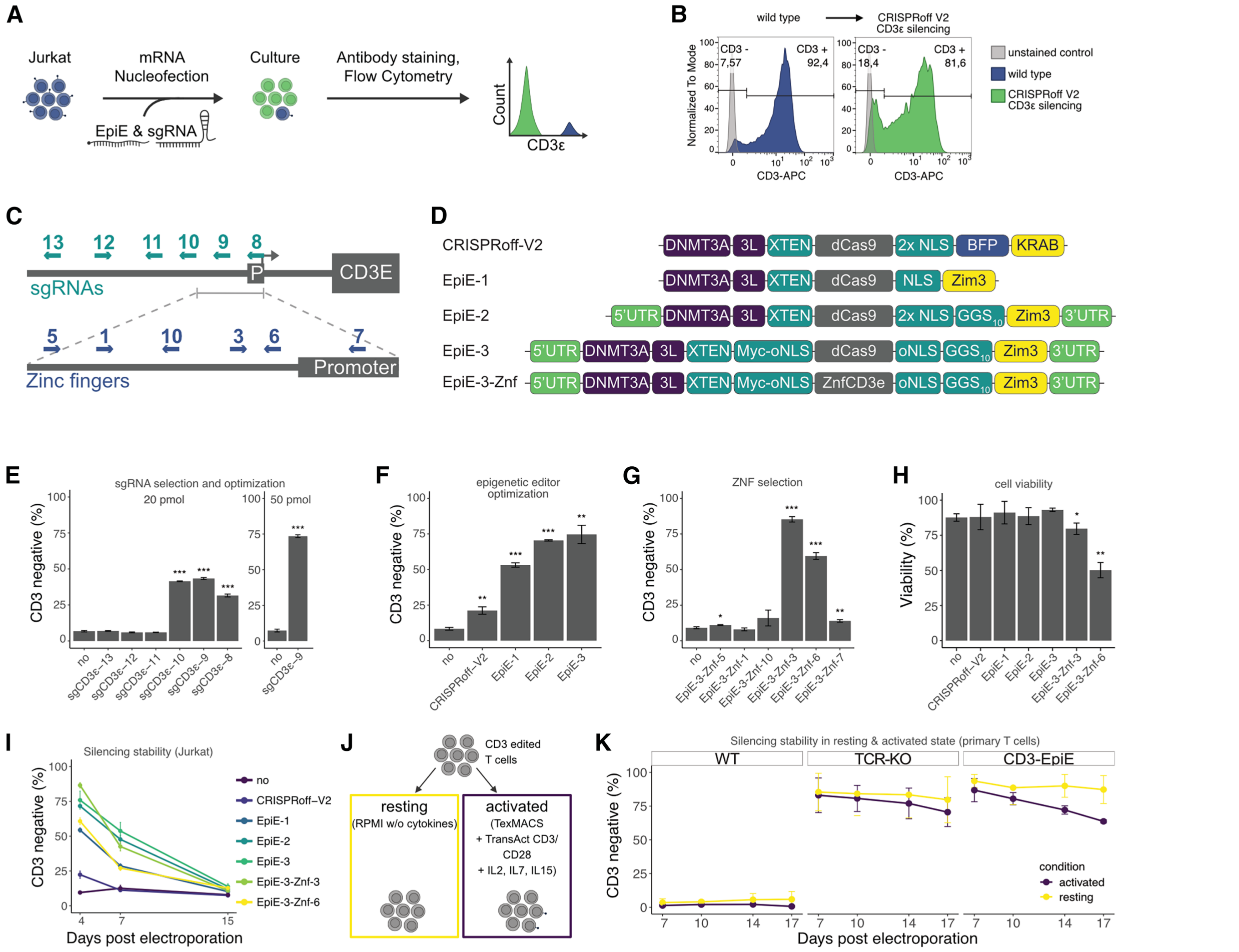

Figure 1. Optimization of epigenetic CD3ε silencing

(A) Illustration of the epigenetic editing and analysis workflow in Jurkat cells. (B) Initial results of CRISPRoff v.2-meditated CD3ε silencing in Jurkat cells by antibody staining and flow cytometry at day 4 post transfection. (C) Schematic of the CD3ε genomic promoter region. Upper line represents the promoter region with gray box labeled 'P' with an arrow, indicating the promoter and the bigger box indicating the first CD3ε exon. The depiction below shows a zoomed in view on the narrower promoter region, in which functional sgRNAs were identified. Designed sgRNAs (*green*) and designer zinc fingers (*blue*) are indicated as arrows with their numbers labeled. (D) Protein domains and features of optimized epigenetic editor constructs and their names. (E) Barplot of flow cytometry data after CD3ε silencing with EpiE-3 and 20 pmol of different sgRNAs and silencing using sgCD3ε-9 with the optimized concentration of 50 pmol. All bar plots represent the mean of three biological replicates, measured at 4 days post electroporation in Jurkat cells and the error bars show their standard deviation (*p* values compared to 'no' control sample: ***< 0.001, ** <0.1, * <0.5). (F) Comparison of CD3ε silencing with 50 pmol of sgCD3ε-9 and the optimized epigenetic editors with CRISPRoff-v.2 as benchmark. (G) CD3ε silencing with 1 pmol mRNA of different zinc finger-based epigenetic editors. (H) Cell viability determined by 4′,6-diamidino-2-phenylindole (DAPI) staining after CD3ε silencing with all epigenetic editor constructs. (I) Time course of CD3ε silencing in Jurkat cells over 17 days. (J) Illustration of the experimental workflow to test silencing durability in primary T cells under resting (unsupplemented RPMI) or activated (TexMACS medium with TransAct CD3/CD28 and IL-2, 7, and 15) conditions. (K) Epigenetic silencing dynamics in activated and resting primary T cells over 17 days. T cells from two independent donors were edited by conventional CRISPR-Cas9 TRAC knockout or epigenetic CD3ε silencing with optimized conditions (EpiE-3/sg CD3ε-9) and the CD3ε silencing monitored under two distinct conditions. T cells from the 'activated' condition were activated 1:500 with TransAct right after electroporation and cultured with IL-2, IL-7, and IL-15 in G-Rex 24 well plates from day 3 post electroporation, allowing for optimal expansion, whereas T cells from the 'resting' condition were not TransAct-activated and cultured in RPMI without interleukins and standard 24 well plates from day 3 post electroporation.

challenges posed by the diverse gene rearrangements of TCRα and TCRβ, making CD3ε a more universal and practical target for epigenetic intervention. Jurkat E6.1 cells, which harbor a stable TCR configuration and are widely used in TCR biology research, were selected as the model system. Epigenetic editors were delivered via mRNA electroporation along with chemically synthesized short guide (sg)RNA and the expression of the TCR complex evaluated by antibody staining and flow cytometry (Figures 1A and S1A). We initially applied the established CRISPRoff-v.2 system to silence the TCR complex by targeting the *CD3ε* promoter in Jurkat cells. This approach achieved moderate silencing efficiency of around 20% TCR depletion (Figure 1B). To improve the efficiency of epigenetic silencing, we designed and tested a number of editing strategies. Multiple sgRNAs and zinc finger proteins were designed to target the

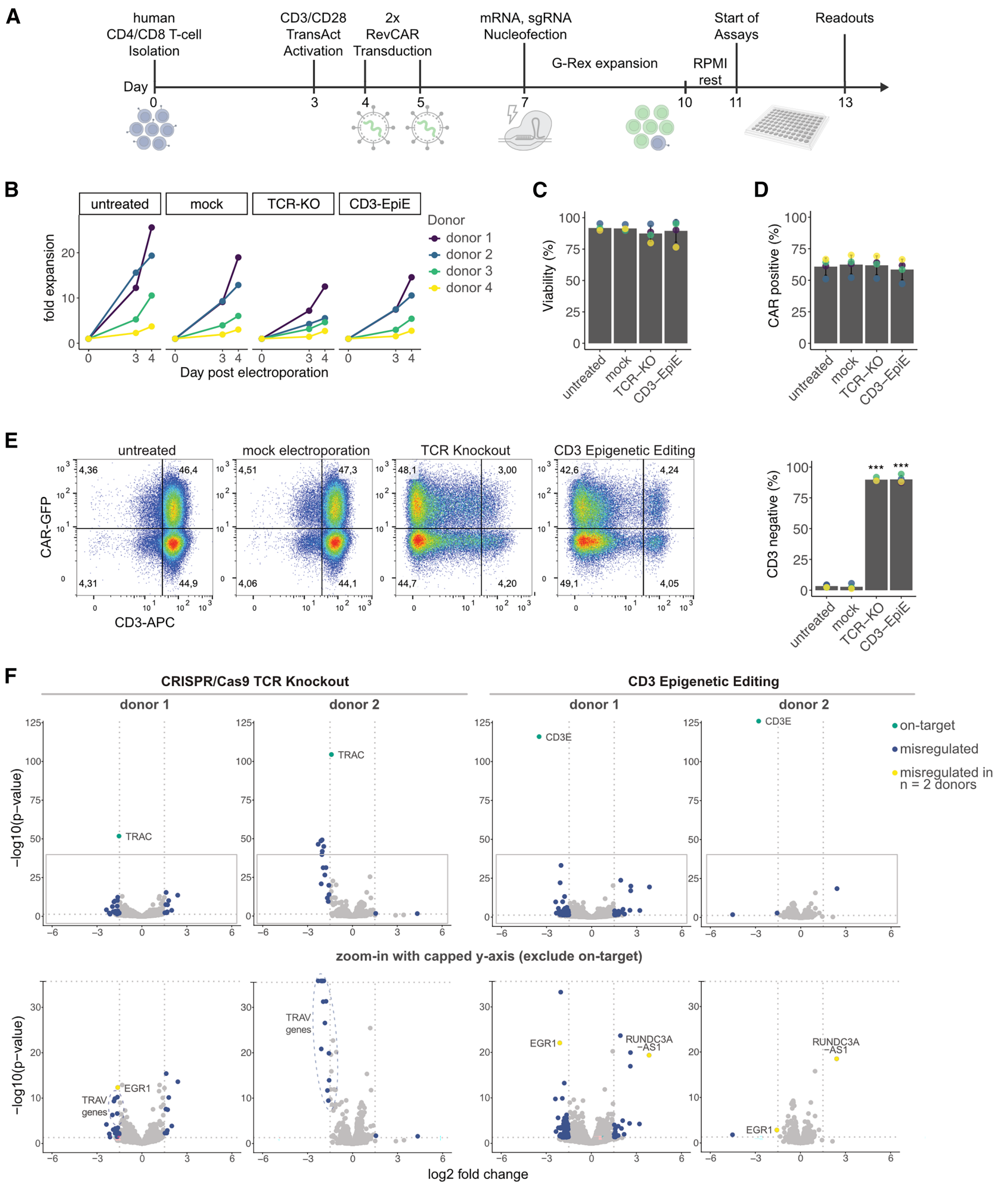

Figure 2. Application in primary RevCAR T cells

(A) Schematic of CAR T production and editing workflow. After the isolation of CD3+ (mixed CD4+/CD8+) healthy donor-derived T cells on day 0, the cells are activated with TransAct and RevCAR-transduced with a lentivirus. Thereafter, CD3ε was epigenetically silenced by electroporation of sgCD3ε-9 and mRNA of EpiE-3 and expanded for 3 days in a G-Rex 24 Well plate in TexMACS supplemented with IL-2, IL-7, and IL-15. The cells were thereafter rested for 24 h in RPMI without interleukins and subsequently subjected to co-culture assays. (B) Expansion curves of RevCAR T cells after electroporation until start of experiments at day 4. RevCAR T Cells from four independent donors are represented as individual lines for each condition (untreated—no electroporation, mock-electroporation with mCherry mRNA, TCR-KO-electroporation with Cas9 mRNA and TRAC-targeting sgRNA, CD3-EpiE—electroporation with EpiE-3 and sgCD3ε-9). (C) Barplot of flow cytometry data of RevCAR T cells from the four independent donors. Viability was assessed by DAPI staining. In all barplots, the bars represent the mean of T cells from four independent donors (*n* = 4), which are individually represented by colored dots and the error bars represent their standard deviation. (D) Barplot of flow cytometry data measuring the RevCAR+ T cell population by EGFP signal intensity. The lentiviral RevCAR construct expresses a RevCAR-T2A-EGFP construct, allowing for an indirect readout. (E) RevCAR+ T cells were determined via EGFP signal and CD3+ T cells by staining with anti-CD3-APC Ab. Representative flow cytometry plots of live RevCAR transduced T cells 4 days post electroporation. Efficiency of CD3ε silencing can

(legend continued on next page)

CD3E promoter region and the epigenetic editor was optimized through several iterations: incorporation of the ZIM3 KRAB domain to promote heterochromatin formation (EpiE-1),[12] addition of hHBB untranslated regions (UTRs) for increased mRNA stability (EpiE-2),[13] and implementation of a high-efficiency nuclear localization signal (hei-tag) to boost nuclear import (EpiE-3)[14] (Figures 1C and 1D). Among the tested sgRNAs, sgCD3ε-8, 9, and 10 showed functional epigenetic silencing (Figure 1E). The evaluation of potential off-target binding sites predicted by the online tool CCTop indicated sgCD3ε-9 as the most specific candidate (Figure S1A).[15] Optimization of the sgRNA concentration showed saturating silencing efficiencies from 50 pmol and boosted the depletion of CD3 to 75% (Figures 1E, S1B, and S1C). Cumulatively, the introduced epigenetic editor optimizations increased silencing efficiency by 3.5-fold (EpiE-3) compared to the original CRISPRoff-v.2 in Jurkat cells (Figure 1F). While the improvement from EpiE-2 to EpiE-3 was not significant at saturating mRNA levels, a direct comparison at limiting mRNA concentrations revealed significant differences, which are important to consider for upscaling (Figure S1D). Even higher efficiencies were achieved when the dCas9 DNA binding domain in EpiE-3 was replaced by one of the *de novo* designed zinc finger domains targeting the *CD3E* promoter (EpiE-3-Znf-3; Figure 1G). However, the zinc finger-based editors led to reduced cell viability compared to the non-toxic dCas9-based editors and were therefore not pursued further (Figure 1H). Taken together, the combination of the optimized epigenetic editor EpiE-3 and sgCD3ε-9 achieved the highest CD3ε silencing efficiency (75%) in Jurkat model cells at 4 days post transfection.

To investigate the long-term dynamic of the edit, Jurkat cells were monitored over an extended time period and showed reversal of the epigenetic silencing form day 7–15 (Figure 1I).

Silencing was next examined in primary T cells, where stable CD3ε suppression was maintained for at least 17 days under resting conditions: following electroporation, T cells were allowed to recover for 3 days in the presence of cytokines and were subsequently cultured in cytokine-free RPMI for the remainder of the experiment (Figures 1H and S2). Conversely, when T cells were activated with the stimulation reagent TransAct immediately following editing and maintained under cytokine stimulation, gene silencing gradually diminished. These findings indicate that CD3ε silencing is stable under low-proliferation conditions, but reverses upon T cell activation and proliferation. To rule out the possibility of preferential expansion of residual wild-type cells, a control of purely CD3 negative T cells was obtained by fluorescence-activated cell sorting (FACS) subsequent to epigenetic silencing (>99% CD3$^-$) and treated in the same way. The control cells similarly returned a CD3 positive population only under activated condition (Figure S2).

In the context of CAR T cell therapy, CAR-induced activation at the tumor site may similarly promote re-expression of the TCR, whereas peripheral resting T cells are likely to maintain TCR silencing. We hypothesized that this balance provides T cells with a unique combination of reduced allogenicity and *in vivo* persistence.

To test the translational potential, we integrated the epigenetic editing approach into a customized CAR T cell production workflow (Figure 2A). T cells from healthy donors were activated, transduced with a lentiviral RevCAR vector,[16] and electroporated with EpiE-3 mRNA and sgCD3ε-9. Edited cells retained RevCAR expression, high viability, and proliferative capacity (Figures 2B–2D). Editing efficiency was robust across donors, with CD3ε silencing reaching up to 92%, confirming the protocol's effectiveness in primary CAR T cells (Figure 2E). RNA-sequencing (RNA-seq) analysis (GEO: GSE299715) was performed on primary T cells from two independent donors in triplicates for the CRISPR-Cas9 KO and CD3 epigenetic silencing condition and untreated cells (Figure 2F; Table S3). Besides the on-target *CD3E*, only two genes were found to be differentially regulated (p value <0.05 and log2 fold change >1.5) after epigenetic editing in all replicates: the transcription factor EGR1 and the lncRNA RUNDC3A-AS1. Both the genes are not in proximity to any possible *in silico* predicted sgCD3ε-9 off-target binding site from CCTop (Table S4), supporting the specificity and safety of the epigenetic editing approach.

To evaluate CAR T cell functionality independently of a fixed tumor antigen and to enable precise control over activation, we applied the editing strategy in combination with the modular RevCAR platform. This adapter-based system uses soluble targeting modules (RevTMs) to direct CAR T cells against a range of antigens, offering flexible retargeting and temporal control. Incorporating a safe, non-genotoxic allogeneic editing approach could further improve the RevCAR system and make it even more universal. For functional validation, we employed the RevCAR-E5B9 targeted to PD-L1-expressing MDA-MB-231 (MDA) breast cancer cells using a soluble PD-L1 RevTM[16] (Figure 3A). RevCAR T cells were co-cultured with luciferase-expressing MDA cells in the presence or absence of RevTM. Epigenetically edited RevCAR T cells demonstrated high cytotoxicity, comparable to untreated and TCR-KO controls (Figure 3B).

be estimated from the *x* axis and CAR+ percentage from the *y* axis with the percentages in each quadrant indicated by numbers. One representative sample is displayed for each treatment and the barplot to the right summarizes the data from all four donors (p values compared to 'untreated' control sample: ***< 0.001). (F) Volcano plot of RNA-seq data after editing of primary T cells from two independent donors in technical triplicates for each treatment (untreated, TCR-KO, and CD3-EpiE). The data from both donors was analyzed separately. The *x* axis represents the average log2 fold change of transcript abundance between untreated and TCR-KO or CD3-EpiE samples. The *y* axis represents the significance (-log10 (p value)) of these changes across the triplicates. Significantly misregulated genes are classified with cut-off values (log2 fold change >1.5; p value <0.05) as indicated by gray dotted lines. The on-target gene, is highlighted in green, other significantly misregulated genes are highlighted in blue and genes that were found to be significantly misregulated in both donors were highlighted in yellow. Top, shows all data points and bottom shows a zoomed-in view with the *y* axis capped at -log10 (p value) = 35, for better resolution and with gene labels.

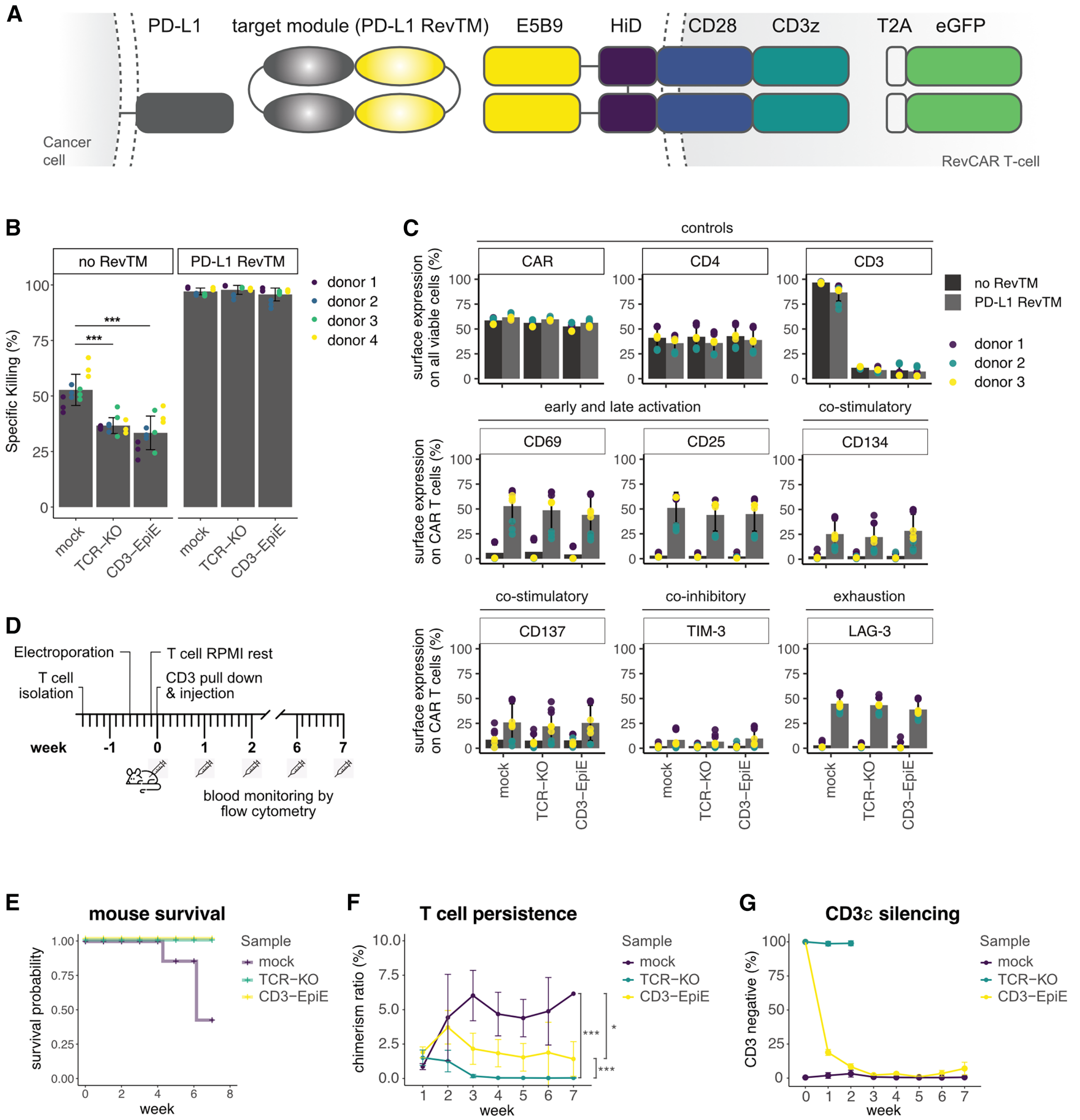

Figure 3. Functional validation upon epigenetic CD3ε silencing *in vitro* and *in vivo*

(A) Schematic representation of the RevCAR construct and its interaction with the target cell. The RevCAR T cells co-expresses eGFP via a T2A. The RevCAR consists of the CD3z activation domain, the CD28 co-stimulatory, transmembrane, and hinge domains and the extracellular E5B9 peptide epitope. A soluble RevTM is required to redirect RevCAR T cells toward cancer cells as it binds to the target cell's surface molecule, such as PD-L1, on cancer cells and simultaneously to the RevCAR-E5B9 T cells. (B) Cytotoxicity assays of RevCAR T cells toward luciferase-expressing MDA-MB-231 cancer cells with or without PD-L1 RevTM. After a co-culture time of 40 h at an E:T ratio of 5:1, the number of residual MDA cancer cells was determined by an luciferase assay. Experiments were conducted with RevCAR T cells from four independent donors ($n = 4$) and three technical replicates. Bars represent the average specific killing, the error bars indicate the standard deviation between replicates donors and colored dots visualize the individual datapoints donors (p values compared to unedited 'mock' sample: ***< 0.001). (C) Barplots of investigated flow cytometry panel after 48 h of co-culture of RevCAR T cells with cancer cells with or without PD-L1 RevTM (gray and black bars, respectively). Experiments were conducted with RevCAR T cells from three independent donors ($n = 3$) and three technical replicates. Bars represent the average percentage of T cells positive for a respective marker, the error bars indicate their standard deviation and colored dots visualize the individual data points from each replicate and donor. (D) Schematic representation of *in vivo* experiment timeline. (E) Kaplan-Meier analysis of survival of mice treated with edited T cells. (F) Persistence of T cells *in vivo*, quantified as chimerism ratio. The chimerism ratio was calculated as the ratio of mouse $CD45^+$ cells to human $CD45^+$ cells as analyzed by flow cytometry from peripheral blood samples. A Mann-Whitney U test was performed to determine statistical significance. (G) Expression of CD3 on human T cells *in vivo*, quantified on human $CD45^+$ cells as analyzed by flow cytometry from peripheral blood samples. Since TCR-knockout T cells did not persist, no data points could be collected after week 2.

Notably, RevCAR-independent background cytotoxicity in the absence of RevTM—presumably mediated via the endogenous TCR due to HLA mismatch—was significantly reduced in TCR/CD3-negative edited T cells, indicating reduced unwanted TCR-mediated alloreactivity of allogeneic T cells through CD3ε silencing or TCR-KO (Figure 3B).

Flow cytometry analysis further confirmed that RevCAR expression, CD4/CD8 ratio, and activation (CD69 and CD25) were unaffected by epigenetic CD3ε editing. All the samples showed RevTM-dependent activation and expression of co-stimulatory (OX40 and 4-1BB) and exhaustion markers (TIM-3 and LAG-3) remained unchanged, indicating that the editing did not alter activation potential or functional phenotype of the RevCAR T cells (Figure 3C).

Finally, primary T cells were injected into NOD scid gamma (NSG) mice and monitored over a period of 7 weeks by antibody staining of peripheral blood samples to investigate their allogenicity and *in vivo* persistence (Figure 3D). While unmodified T cells induced GvHD, epigenetic editing of CD3ε and TCR-KO successfully prevented the onset of GvHD during the monitoring period of 7 weeks, suggesting reduced alloreactivity (Figures 3E and S3). TCR-KO T cells, known to exhibit reduced alloreactivity, were rapidly lost within 3 weeks (Figure 3F). This finding is consistent with the previous reports and highlights the intrinsic challenges of sustaining TCR-KO T cell populations over time.[4] Conversely, T cells treated with epigenetic CD3ε silencing persisted *in vivo* in correlation with a gradual re-expression of surface CD3 on the human T cells over the first 3 weeks (Figures 3F and 3G). These findings indicate that epigenetic silencing could not only avoid the complications of GvHD, but also maintain T cell persistance, which is one critical feature for the success of therapeutic T cells in allogeneic immunotherapy.

DISCUSSION

The development of off-the-shelf allogeneic CAR T cells remains limited by GvHD risk and suboptimal *in vivo* persistence. In this study, we establish an optimized epigenetic editing platform enabling efficient CD3ε silencing. This approach prevented acute GvHD over a 7-week *in vivo* observation period while preserving T cell persistence, addressing two central limitations of current allogeneic CAR T cell strategies.

Beyond conventional CRISPR-Cas9-mediated gene KO, several genome-editing strategies have been developed for allogeneic CAR T cell generation. Cytosine base editors have been used to disrupt splice sites in TRAC, B2M, and PDCD1 through multiplex editing, and similar products have advanced into early phase clinical trials (NCT05885464).[17,18] Prime editing has further enabled duplex disruption of CD3 and B2M, combined with site-specific CAR integration via Bxb1-mediated recombination.[19] While these approaches improve safety relative to nuclease-based editing, the use of nickases still carries a risk of large deletions and chromosomal rearrangements, and base editors have been associated with genome-wide increases in single-nucleotide variants.[1,20] In contrast, epigenetic editing using EpiE-3 represents a fundamentally different strategy, reprogramming gene expression without altering the underlying DNA sequence. This non-disruptive mechanism offers a specific and potentially safer alternative, particularly attractive for clinical translation.

Efficient epigenetic CD3ε silencing was achieved in Jurkat cells and successfully translated to primary T cells and CAR T cells. Transcriptome-wide analysis in primary T cells revealed minimal unintended changes, with only two genes showing significant differential expression. Among these, the lncRNA RUNDC3A-AS1 was upregulated. Although its function in T cells remains poorly characterized, this upregulation is unlikely to represent a direct consequence of off-target epigenetic silencing. The downregulation of EGR1, an immediate-early gene induced by CD3 signaling, is also more plausibly explained by reduced TCR-mediated activation rather than direct epigenetic repression.[21] The fact that EGR1 was also identified as a downregulated transcript in one of the TCR-KO experiments further supports this hypothesis. Future studies would benefit from complementary epigenomic profiling, including single-cell-resolved analyses of chromatin accessibility, histone modifications, and DNA methylation, to identify rare or cell state-specific alterations not detectable by bulk RNA-seq. Given the role of EGR1 in T cell differentiation, monitoring lineage composition following CD3 silencing and TCR-KO will also be informative.[22] Nevertheless, as functionally relevant perturbations are most directly reflected at the transcriptional level, the RNA-seq data presented here provide strong evidence for the epigenetic safety of the EpiE-3 editor.

In vivo, epigenetically silenced T cells showed superior performance compared to TCR-KO cells, persisting throughout the 7-week observation period without inducing acute GvHD, whereas TCR-KO cells were progressively lost. This reduced persistence may, at least in part, reflect the absence of tonic TCR signaling following permanent TCR disruption, which is known to contribute to T cell homeostasis and survival pathways.[23] Notably, CD3ε expression partially re-emerged as early as 2 weeks post-injection, yet animals remained GvHD-free for the remainder of the study, suggesting that low-level or delayed restoration of TCR signaling may be compatible with sustained persistence without triggering overt alloreactivity. Extending the observation period beyond 100 days will be important to assess the impact of clonal expansion and the potential for late-onset GvHD. Nonetheless, within the observed time frame, epigenetic CD3ε silencing achieved a favorable balance between alloreactivity suppression and *in vivo* persistence.

A limitation of the NSG mouse model is the inability to assess host-versus-graft immune rejection, which in clinical settings is commonly addressed by additional edits such as B2M or CIITA disruption.[17,19] In nuclease-based systems, increasing the number of edits proportionally raises the risk of unintended genomic alterations. Epigenetic editing, by contrast, is particularly well suited for multiplex applications, as it avoids cumulative DNA damage. Future studies should therefore explore multiplex epigenetic

targeting to further enhance CAR T cell functionality and evaluate these products in tumor-bearing xenograft models.

In summary, we demonstrate that combining EpiE-3 with sgCD3ε-9 enables efficient silencing of the TCR complex in primary T cells and CAR T cells. This epigenetic editing strategy provides a potentially safer and more flexible alternative to permanent nuclease-mediated TCR-KO, preserving cellular viability, effector function, and *in vivo* persistence. By decoupling alloreactivity control from irreversible genetic disruption, epigenetic silencing may help overcome persistence-related limitations of current allogeneic CAR T cell platforms. Further validation in antigen-driven *in vivo* models will be required to define the therapeutic potential of epigenetically engineered CAR T cells.

MATERIALS AND METHODS

Cell culture

The Jurkat E6.1 wild-type cell line was a kind gift from Dr. Anne Eugster (Bonifacio Laboratory, CRTD, Dresden, Germany) and was cultured in RPMI 1640 medium (Gibco) with 10% fetal bovine serum (FBS) and 1% penicillin/streptomycin. Primary (RevCAR) T cells were cultured in TexMACS (Miltenyi Biotec, Bergisch Gladbach, Germany) supplemented with IL-2, IL-7, and IL-15 (Miltenyi Biotec).

Isolation of primary human T cells and transduction of RevCAR T cells

Primary human T cells were isolated from buffy coats of healthy donors, obtained from the German Red Cross with written consent from volunteers. The local ethics committee of the Medical Faculty Carl Gustav Carus, at the Technische Universität Dresden (Dresden, Germany) approved the research with human T cells (EK138042014). Using density centrifugation with Pancoll solution (1,077 g/mL) (PanBiotech, Aidenbach, Germany), primary T cells were isolated from human peripheral blood mononuclear cells (PBMCs) using a pan T cell isolation kit according to the manufacturer's instructions (Miltenyi Biotec). Isolated T cells were stained with fluorescently labeled mAbs against human CD3 (#130-113-138), CD4 (#130-113-225), CD8 (#130-110-683) (Miltenyi Biotec). T cell lentiviral transduction procedure with RevCARs was done as described previously in supplemented TexMACS.[24]

Electroporation

The Lonza 4D Electroporation SE Cell Line Kit or P3 Primary Kit (Lonza, Basel, Switzerland) was applied for Jurkat E6.1 and primary T cells, respectively. Prior to electroporation, 2×10^5 Jurkat/1×10^6 (RevCAR) T cells were washed with PBS and centrifuged at 400 × g for 5 min/200 × g for 10 min. For one reaction, 16.4 μL of SE Cell Line/P3 Primary Solution was freshly mixed with 3.6 μL Supplement 1, maximally 2 μL of *in vitro* transcribed (IVT) mRNA and chemically synthesized sgRNA (Synthego, Redwood City, USA) added to the solution, the cell pellet resuspended in the mix and transferred to the 20 μL electroporation strip. Electroporation was conducted in the Nucleofector 4D X unit (Lonza), using program CK-116 for Jurkat and DQ-115 for T cells. Thereafter, the cells were quickly recovered by adding 80 μL of pre-warmed supplemented RPMI/TexMACS to the strip and after 5 min incubation, transferred to a 24 well plate with 400 μL of pre-warmed supplemented RPMI or to a G-Rex24 well plate (Wilson Wolf Manufacturing, New Brighton, USA) with 5 mL of pre-warmed supplemented TexMACS, respectively. (RevCAR) T cells were cultured in supplemented TexMACS (Miltenyi Biotec) for 3 days after electroporation, but transferred to RPMI complete medium with 1% penicillin/streptomycin lacking these cytokines 1 day before any functional assay.

sgRNA design and off-target prediction

sgRNAs for epigenetic silencing of CD3ε were designed in a window of 1 kb upstream of the transcriptional start site using CCTop.[15] Predicted sgRNAs with high a high score (>0.75) and high gas chromatography (GC) content were preferentially chosen. Genes affected by potential off-target binding are also provided by the tool and were further enriched with data from the Database of Essential Genes (DEG) on essentiality of the genes.[25]

mRNA IVT

For transient expression of the epigenetic editors and mCherry, mRNA was produced. The coding sequence was amplified from 100 ng of template plasmid using Herculase II Fusion DNA Polymerase (Agilent Technologies, Santa Clara, USA). The mRNA IVT reaction was performed on the purified PCR products using the HiScribe T7 ARCA mRNA Kit with Tailing (New England Biolabs, Ipswich, USA), with partial 5-mCTP and pseudo-UTP (TriLink Biotechnologies, San Diego, USA). Cas9 mRNA was commercially obtained from TriLink.

RNA-seq and analysis

Pellets of 1×10^6 T cells were obtained 4 days post electroporation and RNA isolated using NucleoSpin RNA Kit (Malcherey-Nagel, Düren, Germany) according to the manufacturer's instructions. RNA samples were submitted to mRNA sequencing using NovaSeq X Plus Series (PE150; 9 Gbp per sample) at Novogene (Novogene, Cambridge, UK). The obtained raw reads were processed using standard Cutadapt,[26] RNA STAR[27] and DESeq2[28] workflows. Significantly misregulated genes were defined by a log2 fold change >1.5 and adjusted p value of <0.05.

Cytotoxicity assay

A luminescence-based assay and calculation of specific lysis was performed as previously described.[29] Briefly, 5×10^3 luciferase-expressing MDA-MB-231 cancer cells were co-cultured with RevCAR T cells in effector to target cells (E:T) ratio of 5:1 in the absence or presence of 10 pmol target module (PD-L1 RevTM) per well for a duration of 40 h and luminescence determined using ONE-Glo kit (Promega) in an EnVision microplate reader (PerkinElmer, Waltham, USA).

Activation assay

For duration of 40 h, 5×10^5 RevCAR T cell were co-culture with 1×10^5 MDA-MB-231 cells in the absence or presence of 10 pmol

PD-L1 target module. Thereafter, RevCAR T cells from the three wells were pooled, antibody-stained with antibodies binding to CD3, CD4, CD25, CD69, CD134, CD147, TIM3, LAG3, and eFluor780 Viability Dye in Brilliant Stain Buffer (BD Biosciences) for 30 min at 4°C and measured with LSRFortessa (BD Biosciences).

In vivo experiments

Primary human T cells were electroporated as previously described. After 3 days, the cells were transferred to cytokine free RPMI medium and rested for 24 h. At day 4, the edited T cells (~80% CD3 negative) were purified by CD3 pulldown using EasySep Human CD3 Positive Selection Kit II (Stemcell Technologies), according to manufacturer's instructions. All cells were quality controlled via flow cytometry for CD3 editing and viability (99% CD3 negative, 99% viability). A total of 6×10^5 primary T cells were injected in NSG mice (without tumor xenograft) and peripheral blood samples were analyzed weekly via flow cytometry. Chimerism ratio was calculated as the ratio of viable $mCD45^+$ to ($hCD45^+CD4^+$ and $hCD45^{-}$ $^{+}CD8^+$) cells and CD3 negative percentage only calculated on gated $hCD45^+/CD4^+$ or $CD8^+$). All animal experiments were approved by the Landesdirektion Sachsen, Germany (TVV 27/2025) and performed in accordance with the German and Saxony animal welfare guidelines.

Statistical analysis

Statistical significance was determined as mentioned in figure legends using R. The p values below 0.05 were considered statistically significant ($p \leq 0.05$ (*), $p \leq 0.01$ (**), $p \leq 0.001$ (***). Data are shown as mean values ± SD.

DATA AND CODE AVAILABILITY

The RNA-seq data generated in this study are available in the Gene Expression Omnibus (GEO) repository under accession no. GEO: GSE299715. For other original data, please contact the corresponding author (frank.buchholz@tu-dresden.de).

ACKNOWLEDGMENTS

This work was supported by the BMFTR Cluster4Future project SaxoCell (FZ03ZU1111FA and FZ03ZU2111AB) and the Deutsche Forschungsgemeinschaft, Germany (DFG, project no. 535486506). Flow cytometry analysis of samples from activation assay and *in vivo* experiments in this work were supported by the Flow Cytometry Core Unit, a core facility of the CMCB Technology Platform of the TU Dresden.

AUTHOR CONTRIBUTIONS

P.Y.S. designed and performed the experiments, analyzed data, and wrote the manuscript; A.M.-O. and H.A.S. contributed to experiments; E.C. provided T- and CAR-T cells; R.K. and S.M. conducted *in vivo* experiments; L.L. designed functional CAR T cell assays; A.T. and A.J. provided resources; and F.B. supervised the study, provided funding, and edited the manuscript.

DECLARATION OF INTERESTS

The authors declare no competing financial interests.

SUPPLEMENTAL INFORMATION

Supplemental information can be found online at https://doi.org/10.1016/j.omta.2026.201712.

REFERENCES

1. Fiumara, M., Ferrari, S., Omer-Javed, A., Beretta, S., Albano, L., Canarutto, D., Varesi, A., Gaddoni, C., Brombin, C., Cugnata, F., et al. (2023). Genotoxic effects of base and prime editing in human hematopoietic stem cells. Nat. Biotechnol. *42*, 877–891. https://doi.org/10.1038/s41587-023-01915-4.
2. Nahmad, A.D., Reuveni, E., Goldschmidt, E., Tenne, T., Liberman, M., Horovitz-Fried, M., Khosravi, R., Kobo, H., Reinstein, E., Madi, A., et al. (2022). Frequent Aneuploidy in Primary Human T Cells after CRISPR-Cas9 cleavage. Nat. Biotechnol. *40*, 1807–1813. https://doi.org/10.1038/S41587-022-01377-0.
3. Leibowitz, M.L., Papathanasiou, S., Doerfler, P.A., Blaine, L.J., Sun, L., Yao, Y., Zhang, C.Z., Weiss, M.J., and Pellman, D. (2021). Chromothripsis as an on-target consequence of CRISPR–Cas9 genome editing. Nat. Genet. *53*, 895–905. https://doi.org/10.1038/s41588-021-00838-7.
4. Stenger, D., Stief, T.A., Kaeuferle, T., Willier, S., Rataj, F., Schober, K., Vick, B., Lotfi, R., Wagner, B., Grünewald, T.G.P., et al. (2020). Endogenous TCR promotes in vivo persistence of CD19-CAR-T cells compared to a CRISPR/Cas9-mediated TCR knockout CAR. Blood *136*, 1407–1418. https://doi.org/10.1182/blood.2020005185.
5. Nuñez, J.K., Chen, J., Pommier, G.C., Cogan, J.Z., Replogle, J.M., Adriaens, C., Ramadoss, G.N., Shi, Q., Hung, K.L., Samelson, A.J., et al. (2021). Genome-wide programmable transcriptional memory by CRISPR-based epigenome editing. Cell *184*, 2503–2519.e17. https://doi.org/10.1016/J.CELL.2021.03.025.
6. Cai, R., Lv, R., Shi, X., Yang, G., and Jin, J. (2023). CRISPR/dCas9 Tools: Epigenetic Mechanism and Application in Gene Transcriptional Regulation. Int. J. Mol. Sci. *24*, 14865. https://doi.org/10.3390/IJMS241914865.
7. Galonska, C., Charlton, J., Mattei, A.L., Donaghey, J., Clement, K., Gu, H., Mohammad, A.W., Stamenova, E.K., Cacchiarelli, D., Klages, S., et al. (2018). Genome-wide tracking of dCas9-methyltransferase footprints. Nat. Commun. *9*, 597. https://doi.org/10.1038/s41467-017-02708-5.
8. Policarpi, C., Munafò, M., Tsagkris, S., Carlini, V., and Hackett, J.A. (2024). Systematic epigenome editing captures the context-dependent instructive function of chromatin modifications. Nat. Genet. *56*, 1168–1180. https://doi.org/10.1038/s41588-024-01706-w.
9. O'geen, H., Tomkova, M., Combs, J.A., Tilley, E.K., and Segal, D.J. (2022). Determinants of heritable gene silencing for KRAB-dCas9 + DNMT3 and Ezh2-dCas9 + DNMT3 hit-and-run epigenome editing. Nucleic Acids Res. *50*, 3239–3253. https://doi.org/10.1093/NAR/GKAC123.
10. Call, M.E., and Wucherpfennig, K.W. (2004). Molecular mechanisms for the assembly of the T cell receptor–CD3 complex. Mol. Immunol. *40*, 1295–1305. https://doi.org/10.1016/J.MOLIMM.2003.11.017.
11. Kath, J., Franke, C., Drosdek, V., Du, W., Glaser, V., Fuster-Garcia, C., Stein, M., Zittel, T., Schulenberg, S., Porter, C.E., et al. (2024). Integration of ζ-deficient CARs into the CD3ζ gene conveys potent cytotoxicity in T and NK cells. Blood *143*, 2599–2611. https://doi.org/10.1182/BLOOD.2023020973.
12. Alerasool, N., Segal, D., Lee, H., and Taipale, M. (2020). An efficient KRAB domain for CRISPRi applications in human cells. Nat. Methods *17*, 1093–1096. https://doi.org/10.1038/s41592-020-0966-x.
13. Leppek, K., Byeon, G.W., Kladwang, W., Wayment-Steele, H.K., Kerr, C.H., Xu, A.F., Kim, D.S., Topkar, V.V., Choe, C., Rothschild, D., et al. (2022). Combinatorial optimization of mRNA structure, stability, and translation for RNA-based therapeutics. Nat. Commun. *13*, 1536. https://doi.org/10.1038/s41467-022-28776-w.
14. Thumberger, T., Tavhelidse-Suck, T., Gutierrez-Triana, J.A., Cornean, A., Medert, R., Welz, B., Freichel, M., and Wittbrodt, J. (2022). Boosting targeted genome editing using the hei-tag. eLife *11*, e70558. https://doi.org/10.7554/eLife.70558.
15. Stemmer, M., Thumberger, T., del Sol Keyer, M., Wittbrodt, J., and Mateo, J.L. (2015). CCTop: An Intuitive, Flexible and Reliable CRISPR/Cas9 Target Prediction Tool. PLoS One *10*, e0124633. https://doi.org/10.1371/journal.pone.0124633.
16. Crespo, E., Loureiro, L.R., Stammberger, A., Hoffmann, L., Berndt, N., Hoffmann, A., Dagostino, C., Soto, K.E.G., Rupp, L., Arndt, C., et al. (2025). RevCAR-mediated T-cell response against PD-L1-expressing cells turns suppression into activation. npj Precis. Oncol. *9*, 42. https://doi.org/10.1038/s41698-025-00828-6.

17. Diorio, C., Murray, R., Naniong, M., Barrera, L., Camblin, A., Chukinas, J., Coholan, L., Edwards, A., Fuller, T., Gonzales, C., et al. (2022). Cytosine base editing enables quadruple-edited allogeneic CART cells for T-ALL. Blood *140*, 619–629. https://doi.org/10.1182/BLOOD.2022015825.

18. Webber, B.R., Lonetree, C.L., Kluesner, M.G., Johnson, M.J., Pomeroy, E.J., Diers, M.D., Lahr, W.S., Draper, G.M., Slipek, N.J., Smeester, B.A., et al. (2019). Highly efficient multiplex human T cell engineering without double-strand breaks using Cas9 base editors. Nat. Commun. *10*, 5222. https://doi.org/10.1038/s41467-019-13007-6.

19. Pomeroy, E.J., Anzalone, A.V., Podracky, C.J., Bloch, N.B., Chang, R., Dwivedi, A.A., Laoharawee, K., Wilhelm, A.J., Waterman, D.P., Tedeschi, J.G., et al. (2023). Multiplex Prime Editing and PASSIGE TM for Non-Viral Generation of an Allogeneic CAR-T Cell Product. Blood *142*, 4803. https://doi.org/10.1182/BLOOD-2023-181869.

20. Rao, X., Zhao, H., Shao, C., and Yi, C. (2023). Characterizing off-target effects of genome editors. Curr. Opin. Biomed. Eng. *28*, 100480. https://doi.org/10.1016/J.COBME.2023.100480.

21. Collins, S., Lutz, M.A., Zarek, P.E., Anders, R.A., Kersh, G.J., and Powell, J.D. (2008). Opposing regulation of T cell function by Egr-1/NAB2 and Egr-2/Egr-3. Eur. J. Immunol. *38*, 528–536. https://doi.org/10.1002/EJI.200737157.

22. Lohoff, M., Giaisi, M., Köhler, R., Casper, B., Krammer, P.H., and Li-Weber, M. (2009). Early Growth Response Protein-1 (Egr-1) Is Preferentially Expressed in T Helper Type 2 (Th2) Cells and Is Involved in Acute Transcription of the Th2 Cytokine Interleukin-4. J. Biol. Chem. *285*, 1643. https://doi.org/10.1074/JBC.M109.011585.

23. Harris, M.J., Fuyal, M., and James, J.R. (2021). Quantifying persistence in the T-cell signaling network using an optically controllable antigen receptor. Mol. Syst. Biol. *17*, e10091. https://doi.org/10.15252/MSB.202010091.

24. Feldmann, A., Hoffmann, A., Bergmann, R., Koristka, S., Berndt, N., Arndt, C., Rodrigues Loureiro, L., Kittel-Boselli, E., Mitwasi, N., Kegler, A., et al. (2020). Versatile chimeric antigen receptor platform for controllable and combinatorial T cell therapy. OncoImmunology *9*, 1785608. https://doi.org/10.1080/2162402X.2020.1785608.

25. Luo, H., Lin, Y., Liu, T., Lai, F.L., Zhang, C.T., Gao, F., and Zhang, R. (2021). DEG 15, an update of the Database of Essential Genes that includes built-in analysis tools. Nucleic Acids Res. *49*, D677–D686. https://doi.org/10.1093/NAR/GKAA917.

26. Martin, M. (2011). Cutadapt removes adapter sequences from high-throughput sequencing reads. EMBnet J. *17*, 10. https://doi.org/10.14806/ej.17.1.200.

27. Dobin, A., Davis, C.A., Schlesinger, F., Drenkow, J., Zaleski, C., Jha, S., Batut, P., Chaisson, M., and Gingeras, T.R. (2013). STAR: ultrafast universal RNA-seq aligner. Bioinformatics *29*, 15–21. https://doi.org/10.1093/bioinformatics/bts635.

28. Love, M.I., Huber, W., and Anders, S. (2014). Moderated estimation of fold change and dispersion for RNA-seq data with DESeq2. Genome Biol. *15*, 550. https://doi.org/10.1186/s13059-014-0550-8.

29. Mitwasi, N., Feldmann, A., Bergmann, R., Berndt, N., Arndt, C., Koristka, S., Kegler, A., Jureczek, J., Hoffmann, A., Ehninger, A., et al. (2017). Development of novel target modules for retargeting of UniCAR T cells to GD2 positive tumor cells. Oncotarget *8*, 108584–108603. https://doi.org/10.18632/oncotarget.21017.

OMTA, Volume *34*

Supplemental information

Epigenetic editing balances TCR suppression and persistence in CAR T cells

Pascal Y. Schönberg, Ángela Muñoz-Ovalle, Haidy A. Saleh, Eugenia Crespo, Robert Kuhnert, Susanne Michen, Liliana Loureiro, Achim Temme, Anja Feldmann, and Frank Buchholz

Supplemental Information

Table S1: sgRNA sequences

sgRNA name	Sequence (5' – 3')	Source
sgCD3ε-8	ACAGGAAGTAGAGAGGCCTC	this study
sgCD3ε-9	AGCTGTCCAGACCATGTAGT	this study
sgCD3ε-10	TCTGTGTGGTGGATCTGGCA	this study
sgCD3ε-11	GTGATAGTGTCAAAGTCTGC	this study
sgCD3ε-12	TACCTTAGAGGGTCGTTTTG	this study
sgCD3ε-13	ACTGTACTGTAGTTATATGC	this study

Table S2: Designed zinc finger proteins

Zinc finger name	Predicted DNA target Sequence (5' – 3')	Designed protein sequence	Source
ZnfCD3ε-1	TGTATTTTTCATGGC CCCTCC	FACDICGRKFAHKRSLLDHTRIHTGEKPFACDICGRKFADRS TLRGHTKIHTQRPQIPPKPFACDICGRKFARKDALLNHTRIH TGEKPFACDICGRKFATSSHLGHHTKIHTQRPQIPPKPFACDI CGRKFAWKHVLLNHTRIHTGEKPFACDICGRKFAWLSSLSA HTKIHLRGS	this study
ZnfCD3ε-3	TCAACCAGTCCAGG TGGAGGC	FACDICGRKFARKYHLSQHTRIHTGEKPFACDICGRKFALRW RLNYHTKIHTQRPQIPPKPFACDICGRKFARKWNLLTHTRIH TGEKPFACDICGRKFADPSSLRRHTKIHTQRPQIPPKPFACDI CGRKFADRSTLRNHTRIHTGEKPFACDICGRKFAQSSSLYKH TKIHLRGS	this study
ZnfCD3ε-5	CCAACCTACTACATG GTCTGG	FACDICGRKFARVDVLLDHTRIHTGEKPFACDICGRKFATSS HLSRHTKIHTQRPQIPPKPFACDICGRKFAQSGTLYNHTRIHT GEKPFACDICGRKFALKQTLQRHTKIHTQRPQIPPKPFACDIC GRKFADRSTLRNHTRIHTGEKPFACDICGRKFARRGDLNRH TKIHLRGS	this study
ZnfCD3ε-6	GGAAACGTTCAAGG CAGAGGC	FACDICGRKFARKYHLSQHTRIHTGEKPFACDICGRKFARK WNLRMHTKIHTQRPQIPPKPFACDICGRKFARKDHLKNHTR IHTGEKPFACDICGRKFATSSHLCHHTKIHTQRPQIPPKPFAC DICGRKFADPSNLNRHTRIHTGEKPFACDICGRKFAQRAHLT RHTKIHLRGS	this study
ZnfCD3ε-7	GGGGAGGAGGGTTT CTGAACC	FACDICGRKFADPSNLNRHTRIHTGEKPFACDICGRKFARRS TLRSHTKIHTQRPQIPPKPFACDICGRKFAYKHVLRNHTRIHT GEKPFACDICGRKFAQLYYLSRHTKIHTQRPQIPPKPFACDIC GRKFARKFNLLRHTRIHTGEKPFACDICGRKFARRYSLRCHT KIHLRGS	this study
ZnfCD3ε-10	CAGCCTTCCTAGAAG GCCAAA	FACDICGRKFAQKGNLNSHTRIHTGEKPFACDICGRKFAFRS KLRRHTKIHTQRPQIPPKPFACDICGRKFAQKVHLLNHTRIH TGEKPFACDICGRKFAWKIDLVRHTKIHTQRPQIPPKPFACDI CGRKFALKQTLQSHTRIHTGEKPFACDICGRKFARKWNLSM HTKIHLRGS	this study

Table S3: List of misregulated genes of CD3 epigenetic editing samples from RNAseq

Donor 1			Donor 2		
Gene	log2FoldChange	p-value (adj.)	Gene	log2FoldChange	p-value (adj.)
CD3E	-3,4948277	1,00E-116	CD3E	-2,7924095	0
EGR1	-2,0981087	8,20E-23	EGR1	-1,5674684	0,00141693
RUNDC3A-AS1	3,84431362	4,16E-20	RUNDC3A-AS1	2,40972077	3,06E-19
PSD	-1,8270684	0,04823351	IL3	-4,5201647	0,01561663
PDE4A	-1,7798847	2,60E-06			
EPN2	-1,9848694	0,01650199			
ATP12A	-1,7306593	2,91E-05			
PLXNA2	2,00384955	0,00052638			
MCAM	-1,6447798	0,00809591			

FGFR1	-2,0384521	5,57E-34			
COL5A3	-1,780379	0,00211456			
P2RX5	-1,6489737	1,56E-05			
PITPNM3	-1,9364153	0,0305436			
SORBS1	1,60122999	0,04028409			
MLC1	-1,9297779	1,27E-10			
LYL1	-2,3502757	0,00251292			
PLPPR2	-1,6743729	0,00729399			
ISYNA1	-2,2138477	2,41E-06			
HSPB1	-1,5740675	0,00631777			
AEBP1	-1,5615849	0,00020769			
NPDC1	-1,8593475	0,00013883			
CCL2	3,20270636	5,41E-05			
PCSK4	-1,9529394	0,02902458			
HSD11B1	1,5267058	0,03857889			
EGR1	-2,0981087	8,20E-23			
NUDT10	-2,0432582	0,00030984			
NRSN2	-1,670366	0,00631777			
GAD1	-1,5362809	0,00080276			
CABLES1	-1,7465919	0,00071047			
PCDH8	-1,8443302	5,06E-05			
SCN2A	2,5433992	4,09E-05			
CTSV	1,59538867	0,00065765			
FXYD2	1,61342572	0,00885732			
STON2	1,5531341	0,02466638			
RHOB	-1,8485461	0,03412551			
HES6	-1,5599005	0,00743889			
PLK2	2,0868706	1,01E-05			
DDX4	1,59599097	0,00296725			
CLGN	2,18332199	0,01013801			
SLC5A10	-1,8001748	0,00396074			
NRGN	-2,1098367	0,02195611			
DUSP2	-2,3916972	1,69E-10			
RHPN1	-1,6873771	0,04067628			
NBL1	-1,7292823	0,00151276			
TGFA	1,78938641	0,01855837			
NUAK2	-1,6612045	0,00654461			
COL1A2	1,55575261	0,03364124			
CORO6	-1,7536526	0,00325154			
TNXB	-1,6036959	0,04075605			
DSEL	1,52808341	8,56E-05			
ODAPH	1,78969388	0,00016548			
MARCKSL1	-1,8011079	5,57E-14			
TUBB6	-1,6583386	0,01013801			
SPHK1	-2,0955645	0,02630609			
METRNL	-2,0491371	0,01013801			
RNF212	1,72396032	0,0215462			
EGR3	-2,0514156	0,00049488			
LINC03040	-1,8327786	1,11E-05			
TNFSF15	2,60338448	1,09E-20			
RIMBP3C	-1,5458867	0,01690038			
PDE6G	-1,7573002	9,85E-05			
IFIT1	1,66261324	0,018333			
GAL3ST4	-1,6478281	5,33E-07			
KANK2	-2,1903606	0,00038556			
DLL1	-1,9269118	0,03039918			
MATK	-1,5604297	0,00966267			
IGHM	-1,7938412	0,00029578			
PLXNA4	1,93363359	2,02E-24			
MIR503HG	-1,6563988	0,04779445			
CYP2D8P	-1,9354755	0,04646615			
SGO1-AS1	-1,8027476	0,00342841			
FTH1P23	1,51909084	0,00944042			
SCARF2	-1,9057404	0,02091798			
CHD1-DT	1,62402299	0,01650199			
PURPL	2,5964203	1,08E-17			
LINC03066	-1,9665217	0,00043807			

CIRBP-AS1	-1,5186198	0,03840965			
TIMP2	-1,5603573	5,69E-05			
RIMBP3B	-1,659366	0,00737002			
RIMBP3	-1,8826161	0,0215462			
CCL3	-1,5604053	0,00030893			
GIPR	-1,6583424	3,03E-06			

Table S4: In silico predicted off-target binding sites of sgCD3ε-9

Chromosome	start	end	strand	MM	target_seq	position	gene
chr11	118304525	118304547	-	0	AGCTGTCCAGACCATGTAGT	Exonic	CD3E
chr8	33333326	33333348	+	4	AGGAGGTCAGACCATGTAGT	Intergenic	RNU6-528P
chr2	124917153	124917175	-	3	AGTTGGCCAGAGCATGTAGT	Intergenic	CNTP5
chr2	170863794	170863816	+	3	CACTGTCCAGACCTTGTAGT	Intergenic	GAD1
chr12	93676480	93676502	-	4	GGATGTCTAGAGCATGTAGT	Intergenic	CRADD
chr8	84720378	84720400	+	4	AAATGTCAAGAACATGTAGT	Intronic	RALYL
chr16	77962234	77962256	+	4	AGGGGTCCTGCCCATGTAGT	Intronic	VAT1L
chr3	37496754	37496776	-	4	AGCTGAAAATACCATGTAGT	Intronic	ITGA9
chrX	82111638	82111660	-	4	AGATGCCTAGACTATGTAGT	Intergenic	
chr5	138809578	138809600	+	4	GGCTGTACACATCATGTAGT	Intronic	CTN1
chr18	6836474	6836496	+	4	ATCTGTGCAGTTCATGTAGT	Intronic	ARHGAP28
chr2	12179051	12179073	+	4	AACTGTTCAGGACATGTAGT	Intronic	MIR3681HG
chr6	37882708	37882730	-	4	TGCAGCCCAGACCAGGTAGT	Intronic	RNU1-87P
chr14	103883469	103883491	-	4	GGCTCTCCAGGCCCTGTAGT	Intergenic	CTD-2134A5.4
chr8	39502547	39502569	-	4	AGCTTTTCATACCTTGTAGT	Intronic	ADAM3A
chr16	47777142	47777164	+	4	AACTGTGCAGAGCCTGTAGT	Intronic	CTB-55P19.1
chr4	137092461	137092483	-	4	CTCTCTCCAGACCATGCAGT	Intronic	RP11-138I17.1
chr1	245360680	245360702	+	4	CACTGTCCATACCATCTAGT	Intronic	KIF26B
chr6	63943963	63943985	+	4	TGCTGTGAAGACCATCTAGT	Intronic	EYS
chr10	75138183	75138205	+	4	AGCTTTTCAGACTGTGTAGT	Intronic	SAMD8
chrX	40178387	40178409	+	4	AGATGTTCATACCATATAGT	Intergenic	BCOR
chr10	112686703	112686725	-	4	TTCTGTCCAGAGCATATAGT	Intronic	RP11-25C19.3
chr10	117465139	117465161	+	4	CTGTGTCCAGACCATGTGGT	Intronic	EMX2OS
chr10	85712241	85712263	-	4	ACCTGTACAGAGCATATAGT	Intronic	RP11-93H12.3
chr2	11935562	11935584	+	4	TGCAGTCCAGTCCATGAAGT	Intronic	MIR3681HG
chr7	55736766	55736788	+	4	CCCTGCCCAGACCATGTACT	Exonic	CICP11
chr7	51382271	51382293	+	4	CCCTGCCCAGACCATGTACT	Exonic	CICP17
chr7	55798021	55798043	+	4	CCCTGCCCAGACCATGTACT	Exonic	RP11-419M24.2
chr7	128658782	128658804	-	4	CCCTGCCCAGACCATGTACT	Exonic	CICP14
chr7	39798389	39798411	-	4	CCCTGCCCAGACCATGTACT	Intergenic	CICP22
chrX	30698096	30698118	+	4	ACCTTTCCAGACCAGTTAGT	Intronic	RP11-242C19.2
chr1	53091887	53091909	-	4	AGTTGTGCAAACCATGTCGT	Intronic	SLC1A7
chr5	168975261	168975283	+	4	GGCTCTCCAGAGCATGTGGT	Intronic	CTB-174D11.2
chrX	55163432	55163454	+	4	AGCAATGCAGACCATGTATT	Intergenic	FAM104B
chrX	19325186	19325208	-	4	TGCTGTACAGACCCTGTTGT	Intergenic	PDHA1
chr3	112202780	112202802	-	4	ACCTGACCAGACCATCAAGT	Intronic	SLC9C1
chr2	219413294	219413316	+	4	AGGTGCCCAGACCATTCAGT	Intergenic	DES
chr13	56745239	56745261	-	4	AGTTGCCCAGAACATGTATT	Intergenic	
chr3	149323421	149323443	-	4	ATCTGTGCAGAGCATGTACT	Intronic	TM4SF18
chr10	37967726	37967748	+	4	GGCTGTCAAGACCATTCAGT	Intronic	ZNF25
chr3	142321429	142321451	-	4	AGTTGTCAAGACCATTCAGT	Intronic	XRN1
chr2	169085101	169085123	-	4	AGGTGTCAAGAACATGTATT	Intronic	DHRS9
chr3	84639100	84639122	-	4	GGCTGTAGAGACCATGTAGC	Exonic	LINC00971
chr13	34255433	34255455	+	4	ACCTGTGCAGACAATGTAAT	Intergenic	RP11-282M24.1
chr5	75371301	75371323	-	4	AGTTGTACAGACGATGTATT	Exonic	COL4A3BP
chr22	26805247	26805269	+	4	AGATCTCCAGACCATGGACT	Intergenic	MIATNB
chr3	116335988	116336010	+	4	GGCTGTCAAGACCATGCAAT	Intronic	LSAMP-AS1
chrX	107272279	107272301	+	4	AGATGTCAAGACCATGCAAT	Intergenic	MYCLP1
chr1	234647197	234647219	-	4	AGCTTTCGAGACCATGAATT	Exonic	RP4-781K5.9
chr16	73132574	73132596	-	4	AGCAGACCAGACCATGAAGC	Intronic	C16orf47

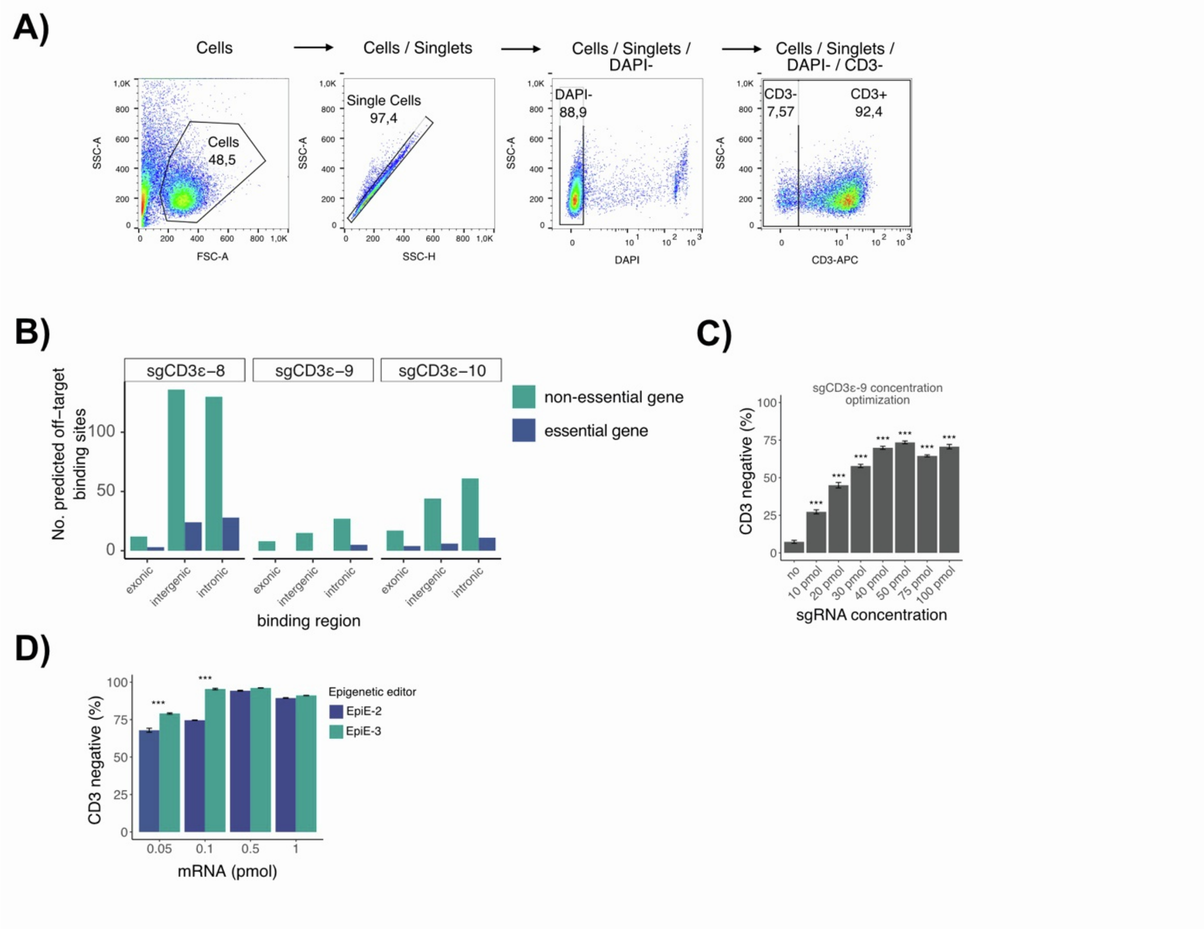

Figure S1: Gating strategy, sgRNA and epigenetic editor optimization

(A) Gating strategy of flow cytometry data to calculate CD3 negative percentage. (B) The number of predicted off-target binding sites in the human genome for the three functional CD3E-targeting sgRNAs is illustrated. Binding sites are specified by the binding region respective to the most proximate gene (exonic, intergenic, intronic) and genes are specified in different colors for their essentiality in human cells according to the Database of Essential Genes (DEG). (C) Optimization of the sgCD3ε-9 concentration for CD3ε silencing with EpiE-3 in Jurkat cells 4 days post transfection (p-values compared to "no" control sample: ***< 0.001). (D) Comparison of the epigenetic editors EpiE-2 and EpiE-3 at low mRNA concentrations in Jurkat cells 4 days post transfection (p-values compared to "EpiE-2" sample at the respective mRNA concentration: ***< 0.001).

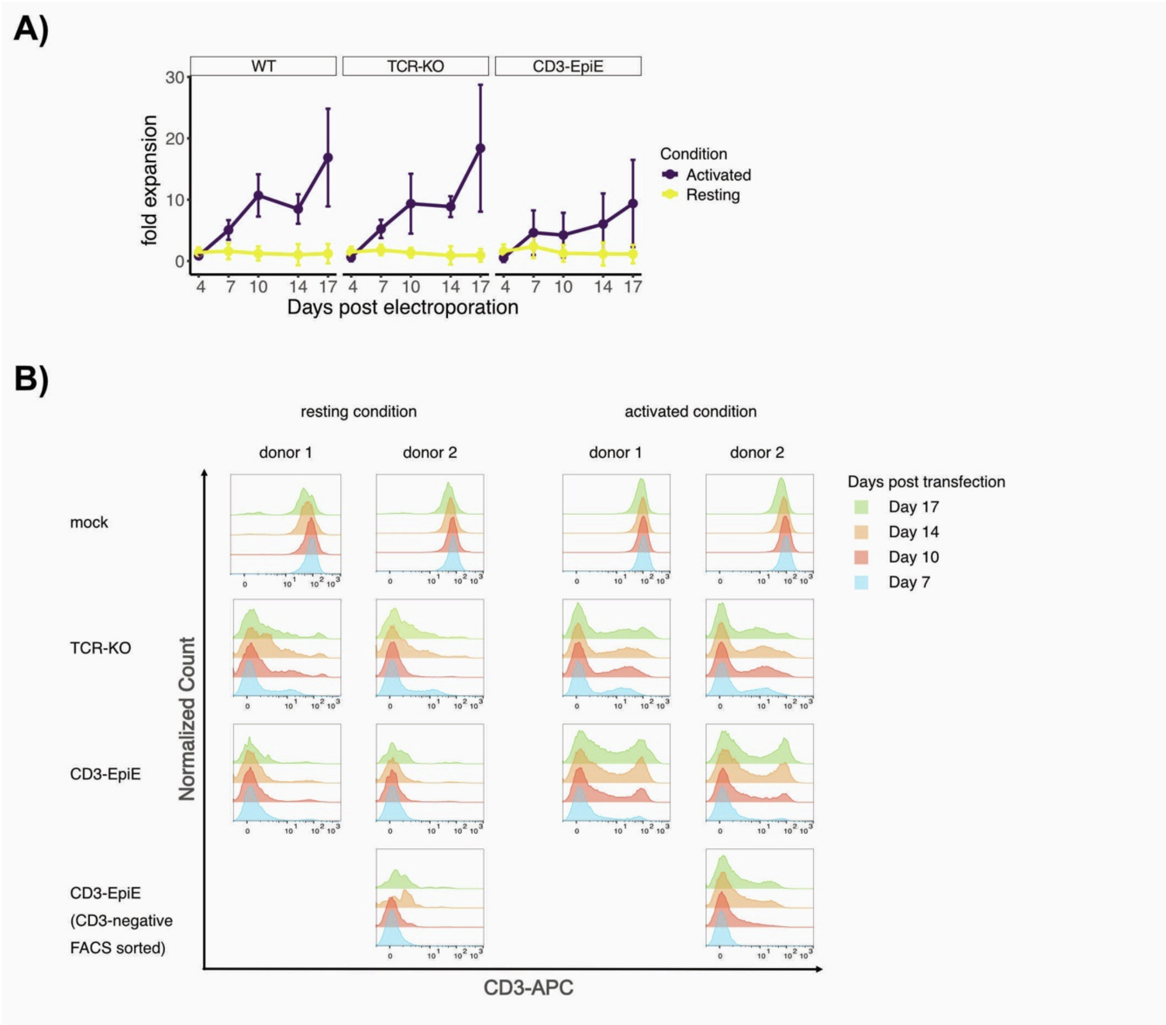

Figure S2: Expansion of primary T cells in resting and activated condition.
(A) Lineplot represents the fold expansion from the starting number of 1 mio. T cells over time in the resting (yellow) and activated (purple) condition over time for all three samples. The experiments was conducted on T cells from two individual donors (n=2). Dots and connecting lines represent the average fold expansion of all three donors and errorbars represent their standard deviation. (B) Flow cytometry histograms for CD3 expression under resting and activated condition for primary T cells from two independent donors. The editing conditions are depicted on the left, activation conditions on top, and timepoints for each sample indicated by different colored histograms in the same plot.

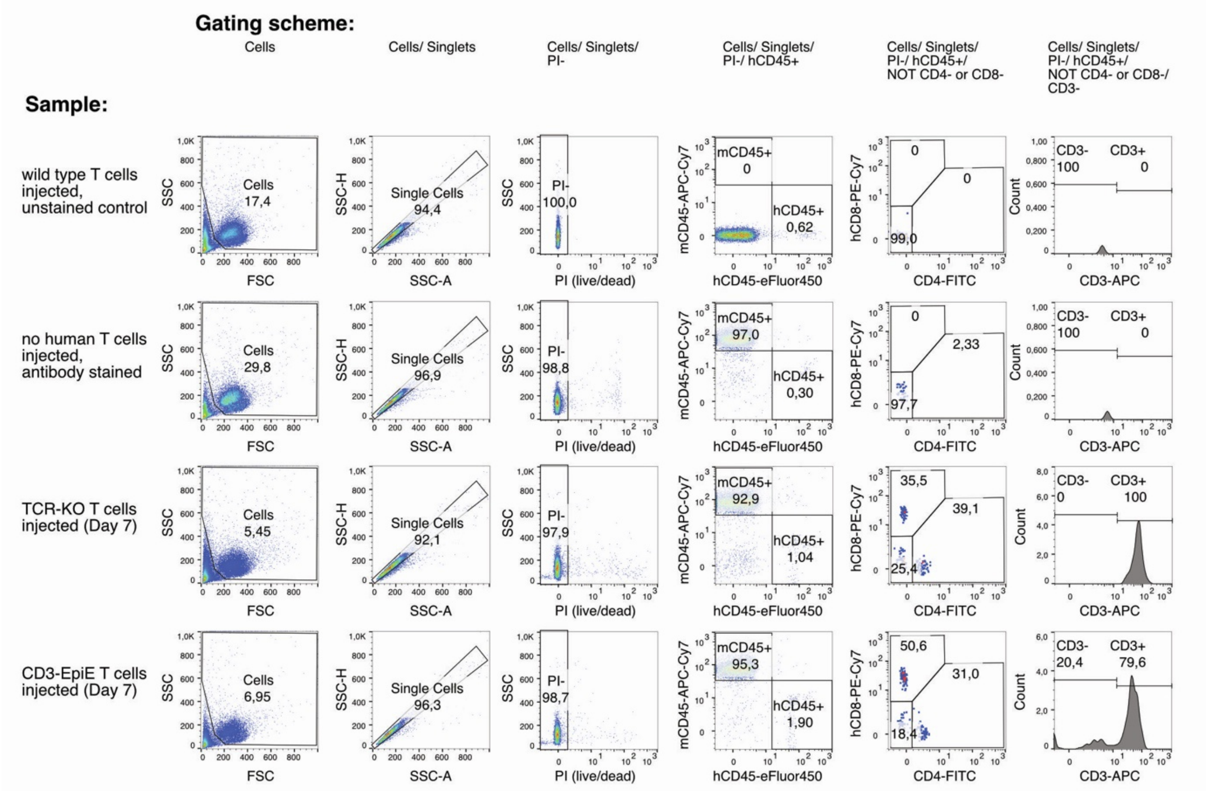

Figure S3: Gating strategy of mouse peripheral blood samples

Gating scheme for the calculation of chimerism ratio and CD3 negative T cells is shown for a unstained control sample, a control sample of peripheral blood without injected human T cells, a sample of injected TCR-KO T cells and injected CD3-EpiE T cells. Chimerism ratio was calculated as the ratio of viable $mCD45^+$ to ($hCD45^+$/ $CD4^+$ and $CD8^+$) cells. The human T cell CD3 negative percentage was estimated on gated Cells/Singlets/PI-/$hCD45^+$/$CD4^+$ and $CD8^+$ cells.
